# Smoking influences the need for surgery in patients with the inflammatory bowel diseases: a systematic review and meta-analysis incorporating disease duration

**DOI:** 10.1186/s12876-016-0555-8

**Published:** 2016-12-21

**Authors:** M. Ellen Kuenzig, Sang Min Lee, Bertus Eksteen, Cynthia H. Seow, Cheryl Barnabe, Remo Panaccione, Gilaad G. Kaplan

**Affiliations:** 1 Department of Medicine, University of Calgary, 3280 Hospital Drive NW, 6D56, Calgary, AB T2N 4N1 Canada; 2 Department of Community Health Sciences, University of Calgary, Calgary, AB Canada; 3 Synder Institute for Chronic Diseases, University of Calgary, Calgary, AB Canada; 4 O’Brien Institute for Public Health, University of Calgary, Calgary, AB Canada

**Keywords:** Crohn’s disease, Ulcerative colitis, Inflammatory bowel disease, Cigarette smoking, Intestinal resection, Colectomy, Survival analysis, Meta-analysis

## Abstract

**Background:**

Studies examining the association between smoking and the need for surgery in patients with Crohn’s disease and ulcerative colitis have reached inconsistent conclusions. These studies often do not differentiate between patients undergoing early surgery and patients having surgery later in their disease course. Our study examined the association between smoking status and time to first bowel resection in patients with Crohn’s disease and ulcerative colitis.

**Methods:**

We searched MEDLINE and EMBASE for studies (*n* = 12) reporting on the association between smoking status (current, former, and never) and surgery in IBD, and incorporated disease duration in the analysis. Hazard ratios (HR) with 95% confidence intervals (CI) were pooled across studies using random effects models.

**Results:**

Current smokers with Crohn’s disease were at increased risk of intestinal resection compared to never smokers (HR 1.27, 95% CI 1.08 to 1.49); however, there was no difference in the need for surgery when comparing former and never smokers (HR 1.11, 95% CI 0.95 to 1.30). In patients with ulcerative colitis, there was no difference in the need for colectomy when comparing current smokers to never smokers (HR 0.98, 95% CI 0.67 to 1.44). Former smokers with ulcerative colitis were at increased risk of colectomy (HR 1.38, 95% CI 1.04 to 1.83) compared to never smokers.

**Conclusions:**

Current smokers with Crohn’s disease are at increased risk of surgery, while former smokers with ulcerative colitis have increased risk of colectomy.

**Electronic supplementary material:**

The online version of this article (doi:10.1186/s12876-016-0555-8) contains supplementary material, which is available to authorized users.

## Background

Cigarette smoking exhibits a paradoxical relationship with the inflammatory bowel diseases (IBD), with smokers being at increased risk of developing Crohn’s disease and at decreased risk of developing ulcerative colitis [1]. Smoking also worsens the prognosis of Crohn’s disease by increasing the risk of penetrating and fibrostenotic complications, as well as the risk of disease recurrence following intestinal resection [2, 3]. Quitting smoking after the diagnosis of Crohn’s disease is associated with a decrease in the risk of a disease flare [4, 5]. However, studies assessing the association between smoking and the need for intestinal resection in patients with Crohn’s disease have reached different conclusions with some demonstrating that smoking increases the risk of intestinal resection and others showing no association [6–14]. Similarly, studies examining the association between smoking and colectomy in patients with ulcerative colitis have demonstrated inconsistent results; some studies suggest that former smokers are more likely to require surgery, while others dispute this association [14–17].

A potential explanation for the heterogeneous results between studies may be the time lapse between diagnosis and first surgery. The need for intestinal resection in patients with Crohn’s disease increases from 16% in the first year following diagnosis to nearly 50% within a decade of diagnosis [18]. Similarly, the proportion of patients with ulcerative colitis requiring colectomy triples from 5% within a year of diagnosis to 16% after 10 years [18]. Thus, accounting for disease duration may be important when studying associations between smoking and surgery in IBD. Disease duration was not evaluated in previous meta-analyses exploring the association between smoking and surgery in IBD [19, 20]. Heterogeneity may also result from differences in the definition of smoking across studies. Many studies dichotomize exposure to smoking, grouping former smokers with current smokers and comparing them to lifetime never smokers. However, due to the differential effects between former and current smokers observed in many observational studies in IBD [21], combining groups of smokers may introduce misclassification bias that could mask important differences between current and former smokers.

Thus, the objective of this systematic review and meta-analysis was to examine the associations between smoking status (current, former, and never) and first intestinal resection, accounting for disease duration in patients with Crohn’s disease and ulcerative colitis.

## Methods

This systematic review and meta-analysis was based on a previously published protocol [22] and is reported in accordance with the Preferred Reporting Items for Systematic Reviews and Meta-analyses (PRISMA) guidelines [23].

### Study identification and selection

A structured literature search was performed using MEDLINE (1946 – April 29, 2016) and EMBASE (1974 – April 29, 2016) databases to identify observational studies examining the association between cigarette smoking and the requirement for first surgery in patients with IBD (Additional file 1: Table S1). We did not restrict the search by language, date of publication, or region of study. We hand searched references of included studies and relevant review articles. The following conference proceedings from gastroenterology meetings are indexed in EMBASE and were included in the search: Digestive Diseases Week 2009–2015; American College of Gastroenterology Annual Scientific Meeting 2010–2015; and Congress of the European Crohn’s and Colitis Organization 2011–2015. Two study authors (MEK and SML) independently screened the full-text abstracts. The kappa statistic was used to evaluate agreement between reviewers. A third author (GGK) resolved disagreements.

Studies were eligible for inclusion if they were: (1) an observational epidemiological study; (2) reported on patients with Crohn’s disease and/or ulcerative colitis; (3) assessed cigarette smoking and separately analyzed current, former, and never smokers; (4) reported on first abdominal surgery (i.e., first intestinal resection) in Crohn’s disease or colectomy in ulcerative colitis; and (5) incorporated disease duration in the analysis using Kaplan-Meier curves and/or hazard ratios (HR) and confidence intervals (CI).

Studies were excluded if (1) they were case series or case reports; (2) assessed the association between smoking and surgery in patients with IBD undetermined (IBD-U) only; (3) reported only on perianal surgeries in patients with Crohn’s disease; (4) were unable to differentiate between first surgery and later surgeries in patients with Crohn’s disease; and (5) reported only on surgeries required for dysplasia or cancer in patients with ulcerative colitis. We additionally excluded studies where current smoking was used as the reference group to ensure the smoking-surgery association was reported consistently across all included studies. When the same cohort of patients was reported in more than one manuscript, we selected the study with the most complete data.

### Data extraction and study quality

Two study authors (MEK and SML) used a standardized data extraction form to independently extract data from eligible studies, including: study design (e.g., source of patients); definitions and source of exposure to cigarette smoke; time to surgery; study location; sample size; and the association between smoking and surgery (e.g., HRs and CIs). The Newcastle-Ottawa Scale was used to assess the quality of individual studies [24]. The scale was adapted to include components of study quality specific to surgical outcomes in IBD (e.g., combining abdominal and perianal surgeries in the definition of surgery in patients with Crohn’s disease) and the assessment of proportional hazards assumption in the statistical analysis.

### Study design and outcomes

Our primary outcomes were: (1) requirement for first intestinal resection in patients with Crohn’s disease; and (2) need for colectomy in patients with ulcerative colitis. Both outcomes were compared for current, former, and never smokers.

### Statistical analysis

Data were analyzed using Stata 12.1 (StataCorp. 2011*. Stata Statistical Software: Release 12*. College Station, TX: StataCorp LP). HRs and 95% CIs were pooled using random effects models to account for expected variability between studies. The most-adjusted HR was used when available. The method proposed by Guyot et al. was used to calculate HRs from Kaplan-Meier curves when HRs were not included in the manuscript [25]. When studies differentiated between heavy and light smokers, the HR comparing heavy smokers to never smokers was used in the meta-analysis. Studies reporting stratified associations between smoking and surgery were pooled using fixed effects analysis prior to being included in the primary analysis.

The I^2^ measure and the Cochran Q statistic were used to assess for heterogeneity with *p* < 0.10 considered statistically significant. We were not able to assess for publication bias due to the small number of studies included [26].

A pre-determined subgroup analysis was based on the method used to identify patients for the study (i.e., population-based cohorts vs. tertiary-care centres). We did not have enough studies to conduct meta-regression to look for differences between subgroups [27]. We conducted the following sensitivity analyses based on the presentation of results in identified studies: (1) replacing the HR comparing heavy smokers to never smokers with the HR for light smokers compared to never smokers; and (2) substituting the age-specific estimates (i.e., 17–40 and >40 years of age at diagnosis) into the pooled analysis, replacing the pooled estimate of the stratum-specific HRs.

## Results

### Description of included studies

The database search identified 2437 records; 2025 records remained after removing duplicates (Additional file 2: Figure S1). The full-text of 327 records were reviewed, including 96 conference abstracts. Eleven studies were eligible for inclusion: seven reported on Crohn’s disease [6–10, 12, 13], three reported on ulcerative colitis [15–17], and one study reported on both Crohn’s disease and ulcerative colitis [14]. One included study was published as a conference abstract [12]. One additional study, reporting on the association between smoking and surgery in Crohn’s disease, was identified after reviewing references of review articles and included studies [11]. Fair agreement was observed between reviewers for assessment of abstracts (κ = 0.34, 95% CI 0.28 to 0.41), and full-text articles (κ = 0.37, 95% CI 0.15 to 0.60). Table 1 (Crohn’s disease) and Table 2 (ulcerative colitis) outline the characteristics of included studies. A list of excluded peer-reviewed manuscripts and reasons for exclusion are provided in Additional file 3: Table S2.

**Table 1:** Characteristics of included studies reporting on the association between smoking and surgery in Crohn’s disease

Study	Country	Source of patients	Definition of smoking	Timing of smoking	Duration of follow-up	Sample size	Adjusted estimates
Deepak 2015 [12]	Unknown	Unknown	Not provided	Unknown	Unknown	79	Age at diagnosis; sex; disease duration; response to treatment
Frolkis 2016 [14]	UK	Population-based	Patient coded in EMR as Current, Former, or Never Smoker within one year of index (diagnosis date)	At diagnosis	Median (Q1, Q3): 5 years (3, 7)	1519	Sex; use of immunosuppressants; steroid use within 90 days of diagnosis^a^
Kariyawasam 2014 [6]	Australia	Tertiary care	Not provided	At diagnosis	Median (Q1, Q3): 11 years (5, 19)	1035	Crude
Lawrance 2013 [7]	Australia; New Zealand	Tertiary care	Current: ≥1 cigarette/day for ≥3 months Former: Smoker who ceased smoking for ≥3 months and had not recommenced prior to the end of follow-up Never: Never regularly smoked ≥1 cigarette/day or had not smoked	At diagnosis	Mean (sd): 17 (9) years	1115	Disease location; perianal disease
Moon 2014 [8]	Korea	Tertiary care	Not provided	At diagnosis	Mean (range): 4 years (0.5, 18)	728	Age at diagnosis; sex; family history of IBD; disease location; disease behaviour; perianal disease
Ng 2016 [13]	Asia (8 countries) and Australia	Population-based	Not provided	At diagnosis	Median (Q1, Q3): 18 months (12, 23)	413	Age; sex; disease behaviour; disease location; perianal disease; treatment within first 3 months; region (Australia vs. Asia)
Peyrin-Biroulet 2012 [9]	USA	Population-based	Not provided	At diagnosis	Median: 12 years	310	Sex; disease location; disease behaviour; need for steroids within 90 days of diagnosis
Renda 2008 [10]	Italy	Tertiary care	Current: ≥7 cigarettes/week for >12 months before diagnosis Former: Smoker who quit ≥1 year before diagnosed and had smoked ≥1 year	At diagnosis	Mean: 6 years	182	Crude
Solberg 2007 [11]	Norway	Population-based^b^	Current: >7 cigarettes/week Former: Years of starting and stopping smoking were compared with years of diagnosis and surgery	At diagnosis	All patients followed for 10 years	237	Crude

*IBD* inflammatory bowel disease, *sd* standard deviation

^a^Hazard ratios were presented separately for young patients (diagnosed between 17 and 40 years of age) and older patients (diagnosed after 40 years of age)

^b^Additional information on study methodology obtained from Moum et al.[31]

**Table 2:** Characteristics of included studies reporting on the association between smoking and colectomy in ulcerative colitis

Study	Country	Source of Patients	Definition of Smoking	Timing of Smoking	Duration of Follow-up	Sample Size	Adjusted Estimates
Beaugerie 2001 [15]	France	Tertiary care	Current: >7 cigarettes/week for ≥6 months after diagnosis Former: Quit for >12 months at time of evaluation	At colectomy or study enrolment	Mean: Current smokers: 10 years Former smokers: 16 years Never smokers: 14 years	96^a^	Crude^b^
Boyko 1988 [16]	USA	HMO	Current: Smoked >100 cigarettes and continued to smoke or initiated smoking after diagnosis Former: Smoked >100 cigarettes and quit prior to diagnosis and did not restart Never: Smoked <100 cigarettes	At diagnosis	Median (range): 8 years (1, 35)	206	Age; sex
Frolkis 2016 [14]	UK	THIN database	Patient coded in EMR as Current, Former, or Never Smoker within one year of index (diagnosis date)	At diagnosis	Median (Q1, Q3): 5 years (3, 8)	3600	Age at diagnosis; sex; use of immunosuppressants; steroid use within 90 days of diagnosis
Hoie 2007 [17]	Europe^c^	Population-based^d^	Current: Maintained same smoking behaviour throughout follow-up Former: Dates when patient started and stopped smoking were compared with year of disease onset and colectomy Never: Patients who had never been daily cigarette smokers^e^	At diagnosis	Median (range): 10 (9, 12) years	771	Crude

*HMO* health maintenance organization

^a^32 former smokers were matched with 32 current smokers and 32 never smokers

^b^Crude hazard ratios were estimated from Kaplan-Meier curves using the method proposed by Guyot et al.[25]

^c^Greece, Israel, Italy, Spain, Denmark, the Netherlands, Norway

^d^Additional information on study methodology obtained from Shivananda et al.[32]

^e^Study also reports a hazard ratio for patients with unknown smoking status relative to individuals who never smoked

### Methodological quality of included studies

The quality of included Crohn’s disease and ulcerative colitis studies is described in Additional file 4: Table S3 and Additional file 5: Table S4, respectively. Only two studies clearly differentiated ulcerative colitis from IBD-U [14, 17]. Five studies recruited patients from tertiary-care centres [6–8, 10, 15], five studies were population-based [9, 11, 13, 14, 17], one study used data from a health maintenance organization (HMO) [16]. The source of patients was unclear in the remaining study [12]. Seven studies that examined the smoking-surgery association in Crohn’s disease were limited to ileocolonic resections [6–9, 11, 13, 14]; one study grouped abdominal and perianal surgeries as a single outcome [10] and this was unclear in the final study [12].

### Smoking and first surgical resection in Crohn’s disease

**Fig. 1:**
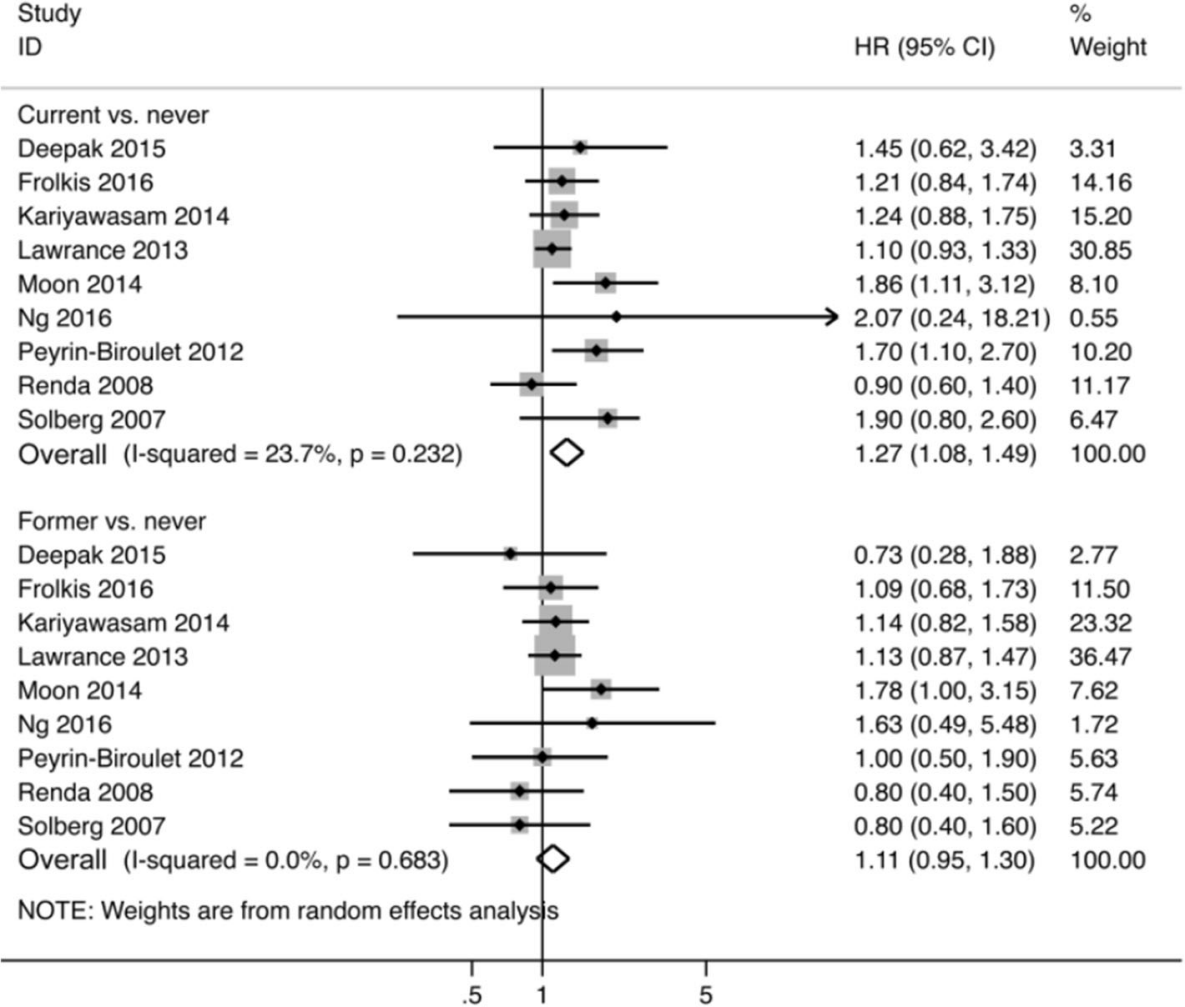
Forest plot depicting the association between smoking and the need for first surgery in patients with Crohn’s disease comparing current to never smokers and former to never smokers

The risk of first surgery was significantly increased in current smokers as compared to never smokers (pooled HR 1.27, 95% CI 1.08 to 1.49, 9 studies; heterogeneity: I^2^ = 24%, *p* = 0.23) (Fig. 1). However, former smokers were not at an increased risk of surgery relative to never smokers (pooled HR 1.11, 95% CI 0.95 to 1.30, 9 studies; heterogeneity: I^2^ = 0%, *p* = 0.60) (Fig. 1). Findings remained consistent when substituting the HR for patients diagnosed between the ages of 17 and 40 and the HR for patients diagnosed after age 40 in the pooled analysis (Additional file 6: Table S5).

One study differentiated between light and heavy current smokers [11]. When the HR comparing light smokers to never smokers was substituted into the meta-analysis, current smoking remained significantly associated with an increased risk of surgery (pooled HR 1.20, 95% CI 1.03 to 1.40, 9 studies; heterogeneity: I^2^ = 18%, *p* = 0.28) (Additional file 7: Figure S2).

Subgroup analysis based on patient recruitment indicated that current smoking increased the risk of surgery in population-based studies (HR 1.48, 95% CI 1.15 to 1.90, 4 studies), but not in patients recruited from tertiary-care centres (HR 1.17, 95% CI 0.94 to 1.46, 4 studies) (Additional file 8: Table S6). Former smokers were not at increased risk of surgery relative to never smokers regardless of the method of patient recruitment.

### Smoking and colectomy in ulcerative colitis

**Fig. 2:**
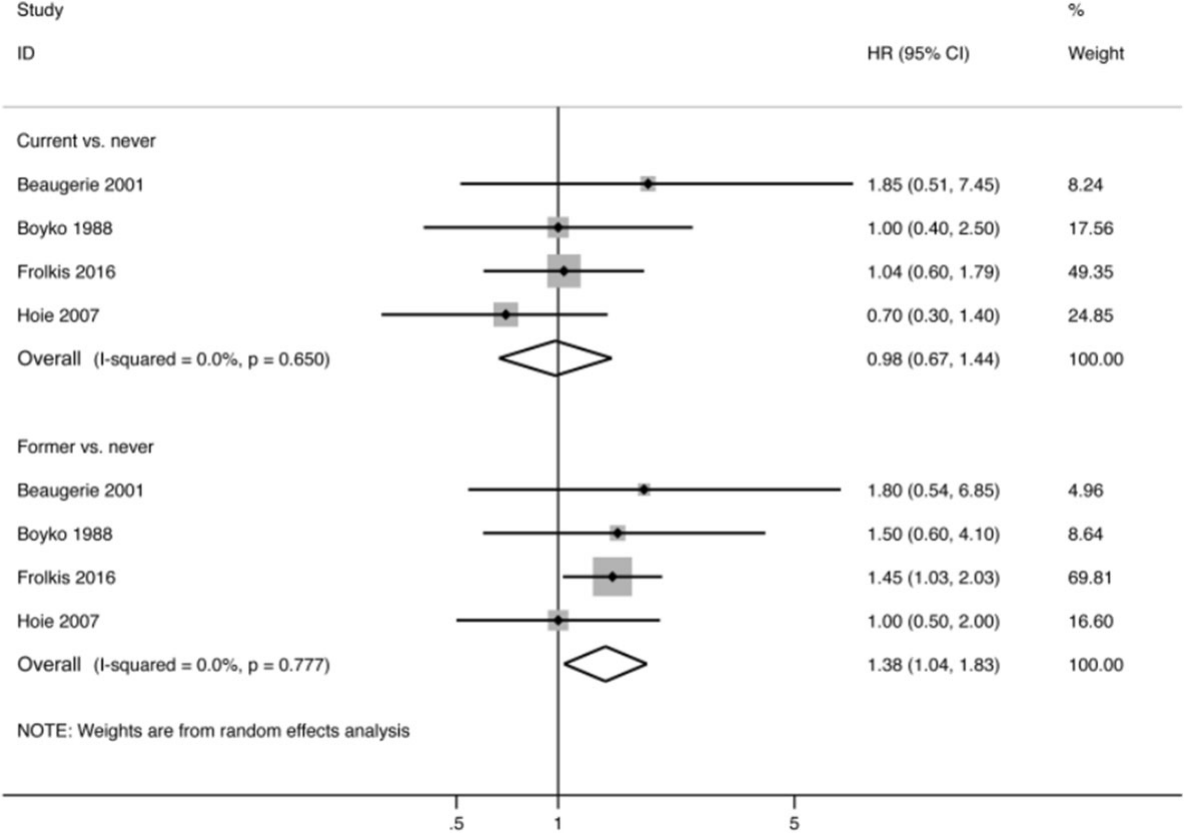
Forest plot depicting the association between smoking and need for colectomy in patients with ulcerative colitis, comparing current to never smokers and former to never smokers

Current smokers were not at increased risk of colectomy compared to never smokers (pooled HR 0.98, 95% CI 0.67 to 1.44, 4 studies; heterogeneity: I^2^ = 0%, *p* = 0.65) (Fig. 2). However, former smokers were significantly more likely to require a colectomy compared to never smokers (pooled HR 1.38, 95% CI 1.04 to 1.83, 4 studies; heterogeneity: I^2^ = 0%, *p* = 0.78) (Fig. 2). When pooling studies reporting on the need for colectomy among patients diagnosed between 17 and 40 years of age, the results remained consistent; however, there was no longer an association between former smoking and the need for colectomy among patients diagnosed with ulcerative colitis after 40 years of age (Additional file 6: Table S5).

Results were consistent in studies in population-based studies and studies based on patients enrolled in an HMO; however, former smoking was no longer associated with the need for colectomy in patients recruited from tertiary-care centres (Additional file 8: Table S6).

## Discussion

This is the first systematic review and meta-analysis of the smoking-surgery association in patients with IBD to incorporate disease duration by including only studies that used time-to-event analyses (Additional file 9). Patients with Crohn’s disease who are current smokers are at increased risk of requiring a first intestinal resection as compared to those who never smoked. In contrast, former smokers were not more likely to need surgery for Crohn’s disease. Patients with ulcerative colitis who were former smokers were at increased risk of colectomy as compared to never smokers. Current smokers were not more likely to require colectomy for ulcerative colitis.

Similar to our findings, a previous meta-analysis assessing the smoking-surgery association in patients with Crohn’s disease demonstrated the increased risk of first surgery among current smokers, but not among former smokers [19]. However, our findings contradict a previous meta-analysis that showed when current and former smokers were combined, smoking decreased the need for colectomy in patients with ulcerative colitis [20]. This dichotomization of smoking status (i.e., combining current and former smokers) occurs in the majority of studies examining the association between smoking and the need for surgery in patients with IBD. However, our study suggests that treating current and former smokers as the same introduces a misclassification bias that blurs the separate effects of current and former smoking on the need for surgery.

We restricted our analysis to studies that accounted for disease duration in order to allow for the potential differential effects of smoking on surgery over time. Studies that do not incorporate disease duration treat patients with IBD who undergo surgery three years following diagnosis in the same fashion as those who will require surgery 15 years after diagnosis. When incorporating time, our meta-analysis showed that former smokers with ulcerative colitis were at increased risk of colectomy compared to never smokers. In contrast, a prior meta-analysis that did not use a time-to-event analysis showed no difference in the odds of colectomy for former and never smokers [28].

Our systematic review identified a paucity of primary studies that examined the effect of more refined definitions of smoking (e.g., dose, duration, and cumulative exposure) or treated smoking as a time-varying covariate. Consequently, we were not able to meaningfully incorporate these elements of smoking into our meta-analysis. These are important considerations because prognosis improves after quitting smoking and the risk of a negative outcome (e.g., disease flare) decreases with time among patients with Crohn’s disease [4, 5]. Future primary studies should consider potential differences between light, moderate, and heavy smokers, as well as potential differences in duration of smoking and cumulative exposure to cigarette smoke (e.g., pack years).

Our systematic review identified one study that stratified the smoking-surgery association by age at diagnosis [14]. This study showed that smoking at diagnosis increased the risk of surgery for Crohn’s disease patients diagnosed after the age of 40, but not for those diagnosed before age 40. In contrast, ulcerative colitis patients diagnosed between the ages of 17 and 40 years and who quit smoking prior to their diagnosis were more likely to undergo a colectomy, but this association was not observed among patients diagnosed with ulcerative colitis after the age of 40 years. In our sensitivity analysis, smoking remained associated with surgery for Crohn’s disease when we included the age-specific risk estimates. In contrast, smoking was only associated with the need for colectomy when we included the risk estimate for patients diagnosed with ulcerative colitis before 40 years of age. Future studies may need to account for age at diagnosis when exploring a relationship between smoking and surgery for IBD.

Although our study provides a comprehensive summary of studies that have investigated the association between smoking and surgery in patients with IBD, it is not without limitations. Firstly, with the exception of disease duration, we did not account for confounding and effect measure modification. The association between smoking and surgery could be influenced by age at diagnosis, disease location, disease behaviour, and/or medications used to treat IBD. Further, ethnic differences may play an additional role on this association, with recent evidence suggesting that the impact of smoking on the development of Crohn’s disease varies across ethnicities [29, 30]. Secondly, selection bias in defining the study populations may have introduced bias in our analyses. For example, the association between time to surgery and current smoking in patients with Crohn’s disease was significant in population-based studies, but not in tertiary-care centres. Patients followed in the community may be at decreased risk of surgery and possibly less likely to be smokers. Thirdly, while we accounted for disease duration, we were unable to analyze the impact of smoking on the need for surgery at a specific time point in the disease course such as at diagnosis or three years following diagnosis.

## Conclusions

After accounting for disease duration, our meta-analysis confirms that cigarette smoking is an important risk factor for surgery in patients with IBD, with current smoking increasing the risk of intestinal resection among patients with Crohn’s disease and former smoking increasing the risk of colectomy among patients with ulcerative colitis. Additionally, our systematic review identified important gaps in the literature. In addition to accounting for disease duration, future studies examining smoking as a risk factor for surgery in patients with IBD should capture detailed smoking information such as dose and duration, and evaluate potential effect measure modification by factors such as age at diagnosis.

## Additional files

Additional file 1: Table S1. Search strategy. (DOCX 84 kb)
Additional file 2: Figure S1. Study flow diagram. (DOCX 108 kb)
Additional file 3: Table S2. List of excluded peer-reviewed manuscripts. (DOCX 301 kb)
Additional file 4: Table S3. Quality of studies assessing the association between smoking and time to first surgery in patients with Crohn’s disease. (DOCX 129 kb)
Additional file 5: Table S4. Quality of studies assessing the association between smoking and colectomy in patients with ulcerative colitis. (DOCX 117 kb)
Additional file 6: Table S5. Sensitivity analysis substituting age-specific smoking-surgery analyses into the overall estimate. (DOCX 75 kb)
Additional file 7: Figure S2. Forest plot depicting the association between smoking and first intestinal resection in patients with Crohn’s disease when substituting the hazard ratio for heavy smoking with that of light smoking. (DOCX 174 kb)
Additional file 8: Table S6. Subgroup analysis based on source of patients with Crohn’s disease and ulcerative colitis. (DOCX 66 kb)
Additional file 9: Raw study Data. Excel file containing hazard ratios from individual studies included in the meta-analysis. (XLSX 45 kb)

## Abbreviations

CI: Confidence interval
HMO: Health maintenance organization
HR: Hazard ratio
IBD: Inflammatory bowel disease
PRISMA: Preferred reporting items for systematic reviews and meta-analyses
SD: Standard deviation
