# Supplementary material for: Smoking influences the need for surgery in patients with the inflammatory bowel diseases: a systematic review and meta-analysis incorporating disease duration
Source: BMC Gastroenterol. 2016 Dec 21;16:143. doi: 10.1186/s12876-016-0555-8 (PMC5178080; doi:10.1186/s12876-016-0555-8)
Supplement: Additional file 1: Table S1. — Search strategy. (DOCX 84 kb) [file 12876_2016_555_MOESM1_ESM.docx]

**Table S1. Search strategy**

| **EMBASE** |
| --- |
| 1. exp Crohn disease/  2. crohn*.tw.  3. exp ulcerative colitis/  4. exp colitis/  5. colitis.tw.  6. exp inflammatory bowel disease/  7. "inflammatory bowel disease*".tw.  8. 1 or 2 or 3 or 4 or 5 or 6 or 7  9. exp smoking/  10. exp smoking cessation/  11. exp smoking cessation program/  12. exp passive smoking/  13. exp smoking habit/  14. exp cigarette smoke/  15. exp maternal smoking/  16. exp parental smoking/  17. exp "smoking and smoking related phenomena"/  18. exp tobacco/  19. exp tobacco dependence/  20. exp tobacco smoke/  21. exp smokeless tobacco/  22. exp tobacco consumption/  23. exp "tobacco use"/  24. smok*.tw.  25. tobacco.tw.  26. cigar*.tw.  27. exp nicotine/  28. nicotine.tw.  29. 9 or 10 or 11 or 12 or 13 or 14 or 15 or 16 or 17 or 18 or 19 or 20 or 21 or 22 or 23 or 24 or 25 or 26 or 27 or 28  30. exp abdominal surgery/  31. exp elective surgery/  32. exp emergency surgery/  33. exp General Surgery/  34. exp major surgery/  35. exp minimally invasive surgery/  36. surger*.tw.  37. surgical*.tw.  38. resecti*.tw.  39. 30 or 31 or 32 or 33 or 34 or 35 or 36 or 37 or 38  40. 8 and 29 and 39 |

| **MEDLINE** |
| --- |
| 1. exp Crohn Disease/  2. crohn*.tw.  3. exp Colitis, Ulcerative/  4. exp Colitis/  5. colitis.tw.  6. exp Inflammatory Bowel Diseases/  7. "inflammatory bowel disease*".tw.  8. 1 or 2 or 3 or 4 or 5 or 6 or 7  9. exp Smoking Cessation/  10. exp Smoking/  11. exp Smoke/  12. smok*.tw.  13. exp Tobacco/  14. exp "Tobacco Use Disorder"/  15. exp Tobacco, Smokeless/  16. exp Tobacco Products/  17. exp Tobacco Smoke Pollution/  18. exp "Tobacco Use"/  19. exp "Tobacco Use Cessation"/  20. exp "Tobacco Use Cessation Products"/  21. exp Nicotine/  22. tobacco.tw.  23. nicotine.tw.  24. cigar*.tw.  25. 9 or 10 or 11 or 12 or 13 or 14 or 15 or 16 or 17 or 18 or 19 or 20 or 21 or 22 or 23 or 24  26. exp Colorectal Surgery/  27. exp General Surgery/  28. surger*.tw.  29. surgical*.tw.  30. resecti*.tw.  31. 26 or 27 or 28 or 29 or 30  32. 8 and 25 and 31 |
