# Supplementary material for: Smoking influences the need for surgery in patients with the inflammatory bowel diseases: a systematic review and meta-analysis incorporating disease duration
Source: BMC Gastroenterol. 2016 Dec 21;16:143. doi: 10.1186/s12876-016-0555-8 (PMC5178080; doi:10.1186/s12876-016-0555-8)
Supplement: Additional file 2: Figure S1. — Study flow diagram. (DOCX 108 kb) [file 12876_2016_555_MOESM2_ESM.docx]

**Figure S1. Study flow diagram**
