# Supplementary material for: Smoking influences the need for surgery in patients with the inflammatory bowel diseases: a systematic review and meta-analysis incorporating disease duration
Source: BMC Gastroenterol. 2016 Dec 21;16:143. doi: 10.1186/s12876-016-0555-8 (PMC5178080; doi:10.1186/s12876-016-0555-8)
Supplement: Additional file 4: Table S3. — Quality of studies assessing the association between smoking and time to first surgery in patients with Crohn’s disease. (DOCX 129 kb) [file 12876_2016_555_MOESM4_ESM.docx]

**Table S3. Quality of studies assessing the association between smoking and time to first surgery in patients with Crohn’s disease**

| Study | Adequate case definition^a^ | Source of smoking information | Source of surgery information | Exclude patients with surgery at diagnosis | Definition of surgery excluded perianal surgeries | Outcome assessors blinded to smoking status | Outcome assessment equivalent for smokers and non-smokers | Loss to follow-up described | Assessed proportional hazards assumption |
| --- | --- | --- | --- | --- | --- | --- | --- | --- | --- |
| Deepak 2015[1] | Unclear | Unclear | Unclear | Unclear | Unclear | Unclear | Unclear | No | Unclear |
| Frolkis 2016  [2] | Yes^b^ | Electronic medical record | Electronic medical record | Unclear | Yes | No^c^ | Unclear^c^ | No | Yes |
| Kariyawasam 2014  [3] | Yes | Chart review | Chart review | Yes | Yes | No | Yes | No | Unclear |
| Lawrance 2013[4] | Yes | Chart Review | Chart Review | Unclear | Yes | No | Yes | No | Unclear |
| Moon 2014  [5] | Yes | Questionnaire; electronic medical record | Questionnaire; electronic medical record | Unclear | Yes | Unclear | Unclear | No | Unclear |
| Ng 2016  [6] | Yes^d^ | Unclear | Unclear | No | Yes | Unclear | Unclear | No | Yes |
| Peyrin-Biroulet 2012  [7] | Yes | Chart review | Chart review | Yes | Yes | No | Yes | No | Unclear |
| Renda 2008  [8] | Yes | Unclear | Unclear | Unclear | No | Unclear | Yes | No | Unclear |
| Solberg 2007[9] | Yes | Interview; chart review | Interview; chart review | Unclear | Yes | Unclear | Yes | Yes | Unclear |

^a^Any study reporting that standard endoscopic, radiologic, or histologic diagnostic criteria were used to identify cases was deemed to have an adequate case definition

^b^Cases of Crohn’s disease and ulcerative colitis were identified using a previously validated list of Read codes

^c^This study use The Health Improvement Network (THIN) database—a collection of records from participating primary care physicians in the UK. Any information on smoking in these patients would have been recorded by the patients’ family physicians and would not have been blinded to clinical information.

^d^Additional information on study methodology obtained from Ng et al[10]

**References**

1. Deepak P, Fletcher JG, Fidler JL, Barlow JM, Sheedy S, Kolbe AB, Harmsen WS, Loftus EV, Hansel SL, Becker BD, Bruining DH: **Radiologic and clinical features as predictors of future hospitalizations for active Crohn“s disease, surgeries, and the need for corticosteroids in patients with small bowel Crohn”s disease**. *Gastroenterology* 2015, **148**:S479–S480.

2. Frolkis AD, de Bruyn J, Jette N, Lowerison M, Engbers J, Ghali W, Lewis JD, Vallerand I, Patten S, Eksteen B, Barnabe C, Panaccione R, Ghosh S, Wiebe S, Kaplan GG: **The association of smoking and surgery in inflammatory bowel disease is modified by age at diagnosis**. *Clin Transl Gastroenterol* 2016, **7**:e165.

3. Kariyawasam VC, Selinger CP, Katelaris PH, Jones DB, McDonald C, Barr G, Chapman G, Colliwshaw J, Lunney PC, Middleton K, Wang RR, Huang T, Andrews J, Leong RW: **Early use of thiopurines or methotrexate reduces major abdominal and perianal surgery in Crohn's disease**. *Inflamm Bowel Dis* 2014, **20**:1382–1390.

4. Lawrance IC, Murray K, Batman B, Gearry RB, Grafton R, Krishnaprasad K, Andrews JM, Prosser R, Bampton PA, Cooke SE, Mahy G, Radford-Smith G, Croft A, Hanigan K: **Crohn's disease and smoking: Is it ever too late to quit?** *J Crohns Colitis* 2013, **7**:e665–e671.

5. Moon CM, Park DI, Kim ER, Kim YH, Lee CK, Lee SH, Kim JH, Huh KC, Jung SA, Yoon SM, Song HJ, Jang HJ, Kim YS, Lee KM, Shin JE: **Clinical features and predictors of clinical outcomes in Korean patients with Crohn's disease: A Korean Association for the Study of Intestinal Diseases multicenter study**. *J Gastroenterol Hepatol* 2014, **29**:74–82.

6. Ng SC, Zeng Z, Niewiadomski O, Tang W, Bell S, Kamm MA, Hu P, de Silva HJ, Niriella MA, Udara WSAAY, Ong D, Ling KL, Ooi CJ, Hilmi I, Lee Goh K, Ouyang Q, Wang YF, Wu K, Wang X, Pisespongsa P, Manatsathit S, Aniwan S, Limsrivilai J, Gunawan J, Simadibrata M, Abdullah M, Tsang SWC, Lo FH, Hui AJ, Chow CM, et al.: **Early course of inflammatory bowel disease in a population-based inception cohort study from 8 countries in Asia and Australia**. *Gastroenterology* 2016, **150**:86–95.e3.

7. Peyrin-Biroulet L, Harmsen WS, Tremaine WJ, Zinsmeister AR, Sandborn WJ, Loftus EV: **Surgery in a population-based cohort of Crohn's disease from Olmsted County, Minnesota (1970-2004)**. *Am J Gastroenterol* 2012, **107**:1693–1701.

8. Renda MC, Orlando A, Civitavecchia G, Criscuoli V, Maggio A, Mocciaro F, Rossi F, Scimeca D, Modesto I, Oliva L, Cottone M: **The role of CARD15 mutations and smoking in the course of Crohn's disease in a Mediterranean area**. *Am J Gastroenterol* 2008, **103**:649–655.

9. Solberg IC, Vatn MH, Høie O, Stray N, Sauar J, Jahnsen J, Moum B, Lygren I: **Clinical course in Crohn’s disease: results of a Norwegian population-based ten-year follow-up study**. *Clin Gastroenterol Hepatol* 2007, **5**:1430–1438.

10. Ng SC, Tang W, Leong RW, Chen M, Ko Y, Studd C, Niewiadomski O, Bell S, Kamm MA, de Silva HJ, Kasturiratne A, Senanayake YU, Ooi CJ, Ling K-L, Ong D, Goh KL, Hilmi I, Ouyang Q, Wang Y-F, Hu P, Zhu Z, Zeng Z, Wu K, Wang X, Xia B, Li J, Pisespongsa P, Manatsathit S, Aniwan S, Simadibrata M, et al.: **Environmental risk factors in inflammatory bowel disease: a population-based case-control study in Asia-Pacific.** *Gut* 2015, **64**:1063–1071.
