# Supplementary material for: Smoking influences the need for surgery in patients with the inflammatory bowel diseases: a systematic review and meta-analysis incorporating disease duration
Source: BMC Gastroenterol. 2016 Dec 21;16:143. doi: 10.1186/s12876-016-0555-8 (PMC5178080; doi:10.1186/s12876-016-0555-8)
Supplement: Additional file 5: Table S4. — Quality of studies assessing the association between smoking and colectomy in patients with ulcerative colitis. (DOCX 117 kb) [file 12876_2016_555_MOESM5_ESM.docx]

**Table S4. Quality of studies assessing the association between smoking and colectomy in patients with ulcerative colitis**

| Study | Adequate case definition^a^ | Excluded patients with IBD-U | Source of smoking information | Source of surgery information | Excluded patients with surgery at diagnosis | Outcome assessors blinded to smoking status | Outcome assessment equivalent for smokers and non-smokers | Loss to follow-up described | Assessed proportional hazards assumption |
| --- | --- | --- | --- | --- | --- | --- | --- | --- | --- |
| Beaugerie 2011  [1] | Yes | Unclear | Chart review | Chart review | Unclear | No | Yes | No | Unclear |
| Boyko 1988  [2] | Yes^b^ | Unclear | Interview | Chart review; administrative database | Unclear | Unclear | Yes | Yes | Not applicable^c^ |
| Frolkis 2016  [3] | Yes^d^ | Yes^e^ | Electronic medical record | Electronic medical record | Unclear | No^f^ | Unclear^f^ | No | Yes |
| Hoie 2007  [4] | Yes | Yes^g^ | Interview | Interview; chart review | Unclear | Unclear | Yes | Yes | Unclear |

Abbreviations: IBD-U, undetermined type of inflammatory bowel disease

^a^Any study reporting that reporting that standard endoscopic, radiologic, or histologic diagnostic criteria were used to identify cases was deemed to have an adequate case definition

^b^Cases identified through an administrative database and verified via chart review

^c^Survival data reported as Kaplan-Meier curves and converted to hazard ratios (HRs) using the method proposed by Guyot et al[5]

^d^Cases of Crohn’s disease and ulcerative colitis were identified using a previously validated list of Read codes

^e^Additional information on study methodology obtained from Lewis et al[6]

^f^This study use The Health Improvement Network (THIN) database—a collection of records from participating primary care physicians in the UK. Any information on smoking in these patients would have been recorded by the patients’ family physicians and would not have been blinded to clinical information.

^g^Additional information on study methodology obtained from Shivananda et al[7]

**References**

1. Beaugerie L, Massot N, Carbonnel F, Cattan S, Gendre JP, Cosnes J: **Impact of cessation of smoking on the course of ulcerative colitis**. *Am J Gastroenterol* 2001, **96**:2113–2116.

2. Boyko EJ, Perera DR, Koepsell TD, Keane EM, Inui TS: **Effects of cigarette smoking on the clinical course of ulcerative colitis**. *Scand J Gastroenterol* 1988, **23**:1147–1152.

3. Frolkis AD, de Bruyn J, Jette N, Lowerison M, Engbers J, Ghali W, Lewis JD, Vallerand I, Patten S, Eksteen B, Barnabe C, Panaccione R, Ghosh S, Wiebe S, Kaplan GG: **The association of smoking and surgery in inflammatory bowel disease is modified by age at diagnosis**. *Clin Transl Gastroenterol* 2016, **7**:e165.

4. Hoie O, Wolters FL, Riis L, Bernklev T, Aamodt G, Clofent J, Tsianos E, Beltrami M, Odes S, Munkholm P, Vatn M, Stockbrugger RW, Moum B: **Low colectomy rates in ulcerative colitis in an unselected European cohort followed for 10 years**. *Gastroenterology* 2007, **132**:507–515.

5. Guyot P, Ades AE, Ouwens MJNM, Welton NJ: **Enhanced secondary analysis of survival data: reconstructing the data from published Kaplan-Meier survival curves.** *BMC Med Res Methodol* 2012, **12**:9.

6. Lewis JD, Brensinger C, Bilker WB, Strom BL: **Validity and completeness of the General Practice Research Database for studies of inflammatory bowel disease.** *Pharmacoepidemiol Drug Saf* 2002, **11**:211–218.

7. Shivananda S, Lennard-Jones J, Logan R, Fear N, Price A, Carpenter L, van Blankenstein M: **Incidence of inflammatory bowel disease across Europe: is there a difference between north and south? Results of the European Collaborative Study on Inflammatory Bowel Disease (EC-IBD).** *Gut* 1996, **39**:690–697.
