# Supplementary material for: Smoking influences the need for surgery in patients with the inflammatory bowel diseases: a systematic review and meta-analysis incorporating disease duration
Source: BMC Gastroenterol. 2016 Dec 21;16:143. doi: 10.1186/s12876-016-0555-8 (PMC5178080; doi:10.1186/s12876-016-0555-8)
Supplement: Additional file 6: Table S5. — Sensitivity analysis substituting age-specific smoking-surgery analyses into the overall estimate. (DOCX 75 kb) [file 12876_2016_555_MOESM6_ESM.docx]

**Table S5. Sensitivity analysis substituting age-specific smoking-surgery analyses into the overall estimate**

|  | **Smoking status** | **HR (95% CI)** | **Heterogeneity** | **Number of studies^a^** |
| --- | --- | --- | --- | --- |
| **Crohn’s disease** | | | | |
| 17 to 40 at diagnosis | Current vs. never | **1.24 (1.03 to 1.50)** | I^2^ = 39%, p = 0.11 | 9 |
|  | Former vs. never | 1.10 (0.94 to 1.29) | I^2^ = 0%, p = 0.67 | 9 |
| > 40 at diagnosis | Current vs. never | **1.43 (1.13 to 1.80)** | **I^2^ = 53%, p = 0.03** | 9 |
|  | Former vs. never | 1.12 (0.95 to 1.32) | I^2^ = 0%, p = 0.66 | 9 |
| **Ulcerative colitis** | | | | |
| 17 to 40 at diagnosis | Current vs. never | 0.76 (0.43 to 1.35) | I^2^ = 27%, p = 0.25 | 4 |
|  | Former vs. never | **1.45 (1.03 to 2.05)** | I^2^ = 0%, p = 0.67 | 4 |
| > 40 at diagnosis | Current vs. never | 1.16 (0.75 to 1.81) | I^2^ = 9%, p = 0.35 | 4 |
|  | Former vs. never | 1.24 (0.87 to 1.77) | I^2^ = 0%, p = 0.84 | 4 |

Abbreviations: CI, confidence interval; HR, hazard ratio

^a^Total number of studies included in the meta-analysis. Only one study (Frolkis et al

[1]) included age-specific associations between smoking and surgery in patients

with Crohn’s disease and ulcerative colitis

**References**

1. Frolkis AD, de Bruyn J, Jette N, Lowerison M, Engbers J, Ghali W, Lewis JD, Vallerand I, Patten S, Eksteen B, Barnabe C, Panaccione R, Ghosh S, Wiebe S, Kaplan GG: **The association of smoking and surgery in inflammatory bowel disease is modified by age at diagnosis**. *Clin Transl Gastroenterol* 2016, **7**:e165.
