# Supplementary material for: Smoking influences the need for surgery in patients with the inflammatory bowel diseases: a systematic review and meta-analysis incorporating disease duration
Source: BMC Gastroenterol. 2016 Dec 21;16:143. doi: 10.1186/s12876-016-0555-8 (PMC5178080; doi:10.1186/s12876-016-0555-8)
Supplement: Additional file 7: Figure S2. — Forest plot depicting the association between smoking and first intestinal resection in patients with Crohn’s disease when substituting the hazard ratio for heavy smoking with that of light smoking. (DOCX 174 kb) [file 12876_2016_555_MOESM7_ESM.docx]

**Figure S2. Forest plot depicting the association between smoking and first intestinal resection in patients with Crohn’s disease when limiting substituting the hazard ratio for heavy smoking with that of light smoking.**
