# Supplementary material for: Smoking influences the need for surgery in patients with the inflammatory bowel diseases: a systematic review and meta-analysis incorporating disease duration
Source: BMC Gastroenterol. 2016 Dec 21;16:143. doi: 10.1186/s12876-016-0555-8 (PMC5178080; doi:10.1186/s12876-016-0555-8)
Supplement: Additional file 8: Table S6. — Subgroup analysis based on source of patients with Crohn’s disease and ulcerative colitis. (DOCX 66 kb) [file 12876_2016_555_MOESM8_ESM.docx]

**Table S6. Subgroup analysis based on source of patients with Crohn’s disease and ulcerative colitis**

| **Smoking Status** | **Subgroup** | **HR (95% CI)** | **Heterogeneity** | **Number of studies** |
| --- | --- | --- | --- | --- |
| **Crohn’s disease** | | | | |
| Current vs. never | Tertiary care | 1.17 (0.94 to 1.46) | I^2^ = 40%, p = 0.17 | 4 |
|  | Population-based or HMO | **1.48 (1.15 to 1.90)** | I^2^ = 0%, p = 0.51 | 4 |
| Former vs. never | Tertiary care | 1.16 (0.94 to 1.42) | I^2^ = 12%, p = 0.33 | 4 |
|  | Population-based or HMO | 1.03 (0.74 to 1.42) | I^2^ = 0%, p = 0.77 | 4 |
| **Ulcerative colitis** | | | | |
| Current vs. never | Tertiary care | 1.85 (0.51 to 7.45) |  | 1 |
|  | Population-based or HMO | 0.93 (0.62 to 1.38) | I^2^ = 0%, p = 0.70 | 3 |
| Former vs. never | Tertiary care | 1.80 (0.54 to 6.85) |  | 1 |
|  | Population-based or HMO | **1.36 (1.02 to 1.82)** | I^2^ = 0%, p = 0.63 | 3 |

Abbreviations: CI, confidence interval; HMO, health maintenance organization; HR, hazard ratio
