# Supplementary material for: Smoking influences the need for surgery in patients with the inflammatory bowel diseases: a systematic review and meta-analysis incorporating disease duration
Source: BMC Gastroenterol. 2016 Dec 21;16:143. doi: 10.1186/s12876-016-0555-8 (PMC5178080; doi:10.1186/s12876-016-0555-8)
Supplement: Additional file 3: Table S2. — List of excluded peer-reviewed manuscripts. (DOCX 301 kb) [file 12876_2016_555_MOESM3_ESM.docx]

**Table S2. List of excluded peer-reviewed manuscripts**

| Study | Reason for exclusion |
| --- | --- |
| Abraham 2003  [1] | Surgery not outcome |
| Aldhous 2007[2] | Current smoking is the reference group |
| Aldhous 2007[3] | Current smoking is the reference group |
| Alikhan 2011  [4] | Review article |
| Almanzar 2012[5] | No time-to-event analysis |
| Alvarez-Lobos 2013[6] | Smoking-surgery association not reported |
| Ananthakrishnan 2013  [7] | Review article |
| Ananthakrishnan 2014  [8] | Review article |
| Ananthakrishnan 2015  [9] | Review article |
| Apostolopoulos 2006  [10] | Review article |
| Are 2011  [11] | Letter to the editor |
| Arnott 2002[12] | Former smokers not analyzed separately |
| Asakura 2012  [13] | Review article |
| Atreja 2014  [14] | Includes patients with prior surgery |
| Attvall 2006[15] | Smoking-surgery association not reported |
| Aujnarain 2013  [16] | Review article |
| Baik 2012  [17] | Review article |
| Balzola 2011[18] | Review article |
| Balzola 2010[19] | Review article |
| Balzola 2011[20] | Review article |
| Balzola 2013[21] | Review article |
| Bastida 2011  [22] | Review article |
| Bege 2009  [23] | Review article |
| Ben-Horin 2009  [24] | Smoking-surgery association not reported |
| Bernklev 2004  [25] | Surgery not outcome |
| Bhat 2009[26] | Smoking-surgery association not reported |
| Biank 2006  [27] | Commentary |
| Bielik 2002  [28] | Descriptive epidemiology |
| Billiet 2014  [29] | Review article |
| Birtwistle 1995[30] | Review article |
| Blonski 2012  [31] | Review article |
| Bossa 2007  [32] | Randomized controlled trial |
| Brant 2003[33] | Former smokers not analyzed separately |
| Breslin 1997[34] | No time-to-event analysis |
| Breuer-Katschinski 1996[35] | Former smokers not analyzed separately |
| Bridger 2012[36] | Former smokers not analyzed separately |
| Butcher 2012  [37] | Surgery not outcome |
| Cabre 2012  [38] | Review article |
| Calabrese 2012  [39] | Case series |
| Camus 2013  [40] | Smoking-surgery association not reported |
| Canas-Ventura 2014  [41] | Insufficient data to pool in meta-analysis |
| Cerqueira 2013  [42] | Review article |
| Cheikh 2002[43] | Former smokers not analyzed separately |
| Chhaya 2015  [44] | Smoking-surgery association not reported |
| Coche 1998  [45] | Review article |
| Cortot 2009  [46] | Review article |
| Cosnes 2002  [47] | Review article |
| Cosnes 2004[48] | Review article |
| Cosnes 2001[49] | Includes patients with prior surgery |
| Cosnes 2002  [50] | Former smokers not analyzed separately |
| Cosnes 1996[51] | Former smokers not analyzed separately |
| Cosnes 2006  [52] | Review article |
| Cosnes 2006  [53] | Smoking-surgery association not reported |
| Cottone 1998  [54] | Review article |
| Coward 2015[55] | Economic analysis |
| Curdia Goncalves 2015  [56] | Smoking-surgery association not reported |
| Da Silva 2015  [57] | Smoking-surgery association not reported |
| Danese 2004  [58] | Review article |
| Dassopoulos 2007[59] | Former smokers not analyzed separately |
| Dassopoulos 2010[60] | Former smokers not analyzed separately |
| De Greef 2011  [61] | Descriptive epidemiology |
| De Saussure 2007  [62] | Surgery not outcome |
| Desmond 2012  [63] | Former smokers not analyzed separately |
| Detry 2003  [64] | Descriptive epidemiology |
| Dhar 2011  [65] | Commentary |
| Dias de Castro 2015  [66] | Smoking-surgery association not reported |
| Dobrowolska-Zachwieja 2005  [67] | No time-to-event analysis |
| Domoslawski 1997  [68] | Conference proceedings |
| Elloumi 2007  [69] | Former smokers not analyzed separately |
| Elloumi 2005  [70] | Combines colonic Crohn’s disease with ulcerative colitis |
| Etienney 2000  [71] | Review article |
| Ferguson 2008  [72] | Smoking-surgery association not reported |
| Fernandez-Blanco Hernaiz 2008  [73] | Review article |
| Fiocchi 2002  [74] | Review article |
| Fischer 2007[75] | Smoking-surgery association not reported |
| Florin 2004  [76] | Former smokers not analyzed separately |
| Forcione 2004  [77] | Cases and controls matched on smoking |
| Fraga 1997  [78] | No time-to-event analysis |
| Frisch 2006  [79] | Editorial |
| Frolkis 2013[80] | Review article |
| Fu 2014[81] | Smoking-surgery association not reported |
| Gaburri 1998  [82] | Descriptive epidemiology |
| Gapasin 2012  [83] | Adherence to treatment guidelines |
| Garcia Rodriguez 2005  [84] | Surgery not outcome |
| Garcia-Paredes 2003  [85] | Editorial |
| Garrido Serrano 2004  [86] | Descriptive epidemiology |
| Gathungu 2013[87] | Provides p-value for *NOD2*-smoking interactions with surgery |
| Goischke 2003  [88] | Review article |
| Goldberg 1993  [89] | Former smokers not analyzed separately |
| Golovics 2013  [90] | Former smokers not analyzed separately |
| Guo 2014[91] | Former smokers not analyzed separately |
| Gustavsson 2013  [92] | Includes patients with prior surgery |
| Halfvarson 2006  [93] | Surgery not outcome |
| Hanauer 1994  [94] | Commentary |
| Hanauer 2012  [95] | Commentary |
| Hanauer 1998  [96] | Review article |
| Hancock 2008[97] | Former smokers not analyzed separately |
| Hansen 2011  [98] | Surgery not outcome |
| Harper 2012  [99] | Former smokers not analyzed separately |
| Hatch 2014  [100] | Former smokers not analyzed separately |
| Henriksen 2007[101] | Smoking-surgery association not reported |
| Hoie 2008  [102] | Smoking-surgery association not reported |
| Hoie 2007  [103] | Surgery not outcome |
| Hovde 2012  [104] | Review article |
| Jiang 2002  [105] | Descriptive epidemiology |
| Jiang 2001  [106] | Descriptive epidemiology |
| Kalkan 2013  [107] | Smoking-surgery association not reported |
| Kanneganti 2013  [108] | Smoking-surgery association not reported |
| Kaplan 2010  [109] | Surgery not outcome |
| Kaplan 2012  [110] | No time-to-event analysis |
| Karczewski 2015  [111] | No time-to-event analysis |
| Karczewski 2014  [112] | No time-to-event analysis |
| Kennelly 2013  [113] | No time-to-event analysis |
| Kim 2012  [114] | Commentary |
| Kiss 2012[115] | Smoking-surgery association not reported |
| Koutroubakis 1996  [116] | Review article |
| Krishnan 2002  [117] | Review article |
| Laghi 2005[118] | Former smokers not analyzed separately |
| Lakatos 2005  [119] | Smoking-surgery association not reported |
| Lakatos 2005  [120] | Former smokers not analyzed separately |
| Lakatos 2009  [121] | Review article |
| Lakatos 2007  [122] | Review article |
| Lakatos 2013  [123] | Former smokers not analyzed separately |
| Law 2013  [124] | Aggregate disease severity outcome |
| Lazarev 2013  [125] | Former smokers not analyzed separately |
| Leiss 2004  [126] | Review article |
| Leong 2006[127] | Smoking-surgery association not reported |
| Leong 2010  [128] | Commentary |
| Lunney 2012  [129] | Review article |
| Lunney 2015  [130] | No time-to-event analysis |
| Manser 2013  [131] | Smoking-surgery association not reported |
| Manser 2016  [132] | Aggregate disease severity outcome |
| Mao 2014  [133] | Former smokers not analyzed separately |
| Margagnoni 2014  [134] | Smoking-surgery association not reported |
| Mazor 2011[135] | Aggregate disease severity outcome |
| McDermott 2013  [136] | Former smokers not analyzed separately |
| Medina 1998  [137] | Former smokers not analyzed separately |
| Mokbel 1998  [138] | Former smokers not analyzed separately |
| Monterubbianesi 2014  [139] | Smoking-surgery association not reported |
| Morgan 2010  [140] | Former smokers not analyzed separately |
| Moum 1997  [141] | Insufficient data to pool in meta-analysis |
| Mozdiak 2015  [142] | Review article |
| Nasir 2013  [143] | No time-to-event analysis |
| Nasir 2013[144] | No time-to-event analysis |
| Ng 2012  [145] | No time-to-event analysis |
| Nuij 2015  [146] | Smoking-surgery association not reported |
| Onal 2014  [147] | Commentary |
| Onali 2009  [148] | First surgery not outcome |
| Parkes 2014  [149] | Review article |
| Perri 1996[150] | Smoking-surgery association not reported |
| Picco 2009  [151] | No time-to-event analysis |
| Pittet 2013[152] | Former smokers not analyzed separately |
| Prosberg 2016  [153] | Aggregate disease severity outcome |
| Puspok 1999  [154] | Smoking-surgery association reported as mean number of surgeries per year |
| Reenaers 2016  [155] | Aggregate disease severity outcome |
| Regueiro 2014[156] | Commentary |
| Rentsch 2006  [157] | Smoking-surgery association not reported |
| Ricciardi 2007[158] | Commentary |
| Riegler 2005  [159] | Former smokers not analyzed separately |
| Ritzmann 2003[160] | Review |
| Romberg-Camps 2009[161] | Aggregate disease severity outcome |
| Russel 1998  [162] | No time-to-event analysis |
| Ryan 2003[163] | Assessed knowledge of impact of smoking on prognosis of Crohn’s disease |
| Safroneeva 2015[164] | Former smokers not analyzed separately |
| Sands 2003[165] | Former smokers not analyzed separately |
| Sato 2015  [166] | Former smokers not analyzed separately |
| Satsangi 1996  [167] | Former smokers not analyzed separately |
| Savoye 2008  [168] | Commentary |
| Seksik 2009[169] | Smoking-surgery association reported as mean number of surgeries per year |
| Seksik 2009  [170] | Smoking-surgery association reported for light and heavy smokers compared to never smokers; former smokers were not analyzed separately |
| Sercombe 2000  [171] | Review |
| Sharma 2013[172] | Includes patients without IBD |
| Sicilia 2005  [173] | No time-to-event analysis |
| Sicilia 2005  [174] | No time-to-event analysis |
| Siddique 2014[175] | Aggregate disease severity outcome |
| Slattery 2011  [176] | Smoking-surgery association not reported |
| Smith 2004  [177] | Smoking-surgery association not reported |
| Solberg 2015  [178] | Smoking-surgery association not reported |
| Song 2011  [179] | Former smokers not analyzed separately |
| Storr 2014  [180] | No time-to-event analysis |
| Strong 2015[181] | Treatment guidelines |
| Szamosi 2010  [182] | Former smokers not analyzed separately |
| Szymanska 2014[183] | No time-to-event analysis |
| Taxonera 2011  [184] | Smoking-surgery association not reported |
| Thapa 2016  [185] | Aggregate disease severity outcome |
| Torres 2012[186] | Descriptive epidemiology |
| Tremaine 2007[187] | No time-to-event analysis |
| Ungar 2013  [188] | Smoking-surgery association not reported |
| Van Assche 2003  [189] | Randomized controlled trial |
| van der Heide 2009  [190] | No time-to-event analysis |
| Vander Cruyssen 2005  [191] | Surgery not outcome |
| Vecchi 2003[192] | Review article |
| Vegh 2014  [193] | Descriptive epidemiology |
| Vester-Andersen 2014[194] | Confidence intervals not reported for hazard ratios |
| Vind 2006  [195] | Descriptive epidemiology |
| Vohra 2000  [196] | Review article |
| Wang 2014  [197] | Gene-environment interactions |
| Waterman 2015  [198] | Aggregate disease severity outcome |
| Yamamoto 1999  [199] | First surgery not outcome |
| Yamamoto 1999[200] | First surgery not outcome |
| Yang 2000[201] | Case series |
| Yarur 2011[202] | Review article |
| Zabana 2013  [203] | No time-to-event analysis |
| Zabana 2013  [204] | Aggregate disease severity outcome |
| Zaharie 2013  [205] | Former smokers not analyzed separately |
| Zaharie 2016  [206] | Former smokers not analyzed separately |
| Zallot 2012  [207] | Review article |
| Zvidi 2013  [208] | Descriptive epidemiology |

**References**

1. Abraham N, Selby W, Lazarus R, Solomon M: **Is smoking an indirect risk factor for the development of ulcerative colitis? An age- and sex-matched case-control study**. *J Gastroenterol Hepatol* 2003, **18**:139–146.

2. Aldhous MC, Drummond HE, Anderson N, Smith LA, Arnott ID, Satsangi J: **Does cigarette smoking influence the phenotype of Crohn's disease? Analysis using the Montreal classification**. *Am J Gastroenterol* 2007, **102**:577–588.

3. Aldhous MC, Drummond HE, Anderson N, Baneshi MR, Smith LA, Arnott ID, Satsangi J: **Smoking habit and load influence age at diagnosis and disease extent in ulcerative colitis**. *Am J Gastroenterol* 2007, **102**:589–597.

4. Alikhan A, Henderson GP, Becker L, Sciallis GF: **Acne treatment and inflammatory bowel disease: What is the evidence?** *J Am Acad Dermatol* 2011, **65**:650–654.

5. Almanzar C, Torres EA: **Lack of association between smoking and surgery for Crohn's disease in Puerto Rico**. *Bol Asoc Med P R* 2012, **104**:66–67.

6. Alvarez-Lobos M, Pizarro DP, Palavecino CE, Espinoza A, Sebastian VP, Alvarado JC, Ibanez P, Quintana C, Diaz O, Kalergis AM, Bueno SM: **Role of Salmonella enterica exposure in Chilean Crohn's disease patients**. *World J Gastroenterol* 2013, **19**:5855–5862.

7. Ananthakrishnan AN: **Environmental risk factors for inflammatory bowel disease**. *Gastroenterol Hepatol (N Y)* 2013, **9**:367–374.

8. Ananthakrishnan AN: **Environmental risk factors for inflammatory bowel diseases: a review**. *Dig Dis Sci* 2014, **60**:290–298.

9. Ananthakrishnan AN: **Epidemiology and risk factors for IBD**. *Nat Rev Gastroenterol Hepatol* 2015, **12**:205–217.

10. Apostolopoulos P: **Environmental factors in IBD**. *Ann Gastroenterol* 2006, **19**:152–154.

11. Are C, Ly Q, Sasson AR: **Primum non nocere, or leave that ostomy alone**. *Arch Surg* 2011, **146**:99–100.

12. Arnott ID, Williams N, Drummond HE, Ghosh S: **Whole gut lavage fluid interleukin-1beta and interleukin-8 in smokers and non-smokers with Crohn's disease in clinical remission**. *Dig Liver Dis* 2002, **34**:424–429.

13. Asakura K: **[Etiology of inflammatory bowel disease--lifestyle and environmental risk factors]**. *Nihon Rinsho* 2012, **70**:73–78.

14. Atreja A, Aggarwal A, Dwivedi S, Rieder F, Lopez R, Lashner BA, Brzezinski A, Vargo JJ, Shen B: **Safety and efficacy of endoscopic dilation for primary and anastomotic Crohn's disease strictures**. *J Crohns Colitis* 2014, **8**:392–400.

15. Attvall E, Frigyesi A, Sternby B: **What is the impact of resistance to activated protein C (Leiden mutation to factor V) in inflammatory bowel disease?** *Int J Colorectal Dis* 2006, **21**:705–710.

16. Aujnarain A, Mack DR, Benchimol EI: **The role of the environment in the development of pediatric inflammatory bowel disease topical collection on pediatric gastroenterology**. *Curr Gastroenterol Rep* 2013, **15**:326.

17. Baik SH, Kim WH: **A comprehensive review of inflammatory bowel disease focusing on surgical management**. *J Korean Soc Coloproctol* 2012, **28**:121–131.

18. Balzola F, Bernstein C, Ho GT: **The inflammatory bowel diseases and ambient air pollution: a novel association**. *Inflamm Bowel Dis Monit* 2011, **11**:163–164.

19. Balzola F, Bernstein C, Ho GT, Lees C: **Natural history of Crohn's disease: comparison between childhood- and adult-onset disease**. *Inflamm Bowel Dis Monit* 2010, **11**:84.

20. Balzola F, Bernstein C, Ho GT, Lees C: **Natural history of Crohn's disease in a population-based cohort from Cardiff (1986-2003): a study of changes in medical treatment and surgical resection rates**. *Inflamm Bowel Dis Monit* 2011, **11**:121–122.

21. Balzola F, Cullen G, Ho GT, Russell RK: **The role of the environment in the development of pediatric inflammatory bowel disease**. *Inflamm Bowel Dis Monit* 2013, **14**:31–32.

22. Bastida G, Beltran B: **Ulcerative colitis in smokers, non-smokers and ex-smokers**. *World J Gastroenterol* 2011, **17**:2740–2747.

23. Bege T, Berdah SV, Moutardier V, Brunet C: **Risks related to tobacco use in general and intestinal surgery. [French] Risques lies au tabagisme en chirurgie generale et digestive**. *J Chir (Paris)* 2009, **146**:532–536.

24. Ben-Horin S, Avidan B, Yanai H, Lang A, Chowers Y, Bar-Meir S: **Familial clustering of Crohn's disease in Israel: prevalence and association with disease severity**. *Inflamm Bowel Dis* 2009, **15**:171–175.

25. Bernklev T, Jahnsen J, Aadland E, Sauar J, Schulz T, Lygren I, Henriksen M, Stray N, Kjellevold O, Vatn M, Moum B: **Health-related quality of life in patients with inflammatory bowel disease five years after the initial diagnosis**. *Scand J Gastroenterol* 2004, **39**:365–373.

26. Bhat M, Nguyen GC, Pare P, Lahaie R, Deslandres C, Bernard EJ, Aumais G, Jobin G, Wild G, Cohen A, Langelier D, Brant S, Dassopoulos T, McGovern D, Torres E, Duerr R, Regueiro M, Silverberg MS, Steinhart H, Griffiths AM, Elkadri A, Cho J, Proctor D, Goyette P, Rioux J, Bitton A: **Phenotypic and genotypic characteristics of inflammatory bowel disease in French Canadians: comparison with a large North American repository**. *Am J Gastroenterol* 2009, **104**:2233–2240.

27. Biank VF, Kugathasan S: **What are the risk factors for surgery and their implications for management of children with Crohn's disease?** *Nat Clin Pract Gastroenterol Hepatol* 2006, **3**:660–661.

28. Bielik J, Hlista M, Cernok S, Sorf M: **Clinical and drug profile of morbus Crohn. [Slovak] Klinicky a liekovy profil morbus Crohn**. *Ceska a Slovenska Gastroenterologie a Hepatologie* 2002, **56**:158–163.

29. Billiet T, Ferrante M, Van Assche G: **The use of prognostic factors in inflammatory bowel diseases**. *Curr Gastroenterol Rep* 2014, **16**:1–14.

30. Birtwistle J: **Quitting smoking could increase your risk of surgery for ulcerative colitis!** *Br J Theatre Nurs* 1995, **5**:27–28.

31. Blonski W, Buchner AM, Lichtenstein GR: **Clinical predictors of aggressive/disabling disease: ulcerative colitis and Crohn disease**. *Gastroenterol Clin North Am* 2012, **41**:443–462.

32. Bossa F, Fiorella S, Caruso N, Accadia L, Napolitano G, Valvano MR, Andriulli A, Annese V: **Continuous infusion versus bolus administration of steroids in severe attacks of ulcerative colitis: a randomized, double-blind trial**. *Am J Gastroenterol* 2007, **102**:601–608.

33. Brant SR, Picco MF, Achkar JP, Bayless TM, Kane SV, Brzezinski A, Nouvet FJ, Bonen D, Karban A, Dassopoulos T, Karaliukas R, Beaty TH, Hanauer SB, Duerr RH, Cho JH: **Defining complex contributions of NOD2/CARD15 gene mutations, age at onset, and tobacco use on Crohn's disease phenotypes**. *Inflamm Bowel Dis* 2003, **9**:281–289.

34. Breslin NP, McDonnell C, O'Morain C: **Surgical and smoking history in inflammatory bowel disease: a case-control study**. *Inflamm Bowel Dis* 1997, **3**:1–5.

35. Breuer-Katschinski BD, Hollander N, Goebell H: **Effect of cigarette smoking on the course of Crohn's disease**. *Eur J Gastroenterol Hepatol* 1996, **8**:225–228.

36. Bridger S, Lee JC, Bjarnason I, Jones JE, Macpherson AJ: **In siblings with similar genetic susceptibility for inflammatory bowel disease, smokers tend to develop Crohn's disease and non-smokers develop ulcerative colitis**. *Gut* 2002, **51**:21–25.

37. Butcher RO, Nixon E, Sapundzieski M, Filobbos R, Limdi JK: **Radiation exposure in patients with inflammatory bowel diseaseprimum non nocere?** *Scand J Gastroenterol* 2012, **47**:1192–1199.

38. Cabre E, Domenech E: **Impact of environmental and dietary factors on the course of inflammatory bowel disease**. *World J Gastroenterol* 2012, **18**:3814–3822.

39. Calabrese E, Yanai H, Shuster D, Rubin DT, Hanauer SB: **Low-dose smoking resumption in ex-smokers with refractory ulcerative colitis**. *J Crohns Colitis* 2012, **6**:756–762.

40. Camus M, Seksik P, Bourrier A, Nion-Larmurier I, Sokol H, Baumer P, Beaugerie L, Cosnes J: **Long-term outcome of patients with crohn's disease who respond to azathioprine**. *Clin Gastroenterol Hepatol* 2013, **11**:389–394.

41. Canas-Ventura A, Marquez L, Ricart E, Domenech E, Gisbert JP, Garcia-Sanchez V, Marin-Jimenez I, Rodriguez-Moranta F, Gomollon F, Calvet X, Merino O, Vazquez-Romero N, Esteve M, Iborra M, Gutierrez A, Vera M, Andreu M, PanÉS J, Garcia V, Penalva M, Garcia-Planella E, Vera I, Nos P, Vazquez N, Gento E, Fernandez-Salazar L, Barrio J, Martin MD, Munoz F, Ponferrada A, et al.: **Risk of colectomy in patients with ulcerative colitis under thiopurine treatment**. *J Crohns Colitis* 2014, **8**:1287–1293.

42. Cerqueira RM, Lago PM: **Clinical factors predictive of Crohn's disease complications and surgery**. *Eur J Gastroenterol Hepatol* 2013, **25**:129–134.

43. Cheikh I, Ben Ammar A, Essid M, Azzouz M, Ettahri N, Krichene M, Bouzaidi S, Ennajar T: **[Treatment and outcome of Crohn's disease without initial complications. Results of a retrospective, multicenter Tunisian study]**. *Tunis Med* 2002, **80**:193–198.

44. Chhaya V, Pollok RCG, Cecil E, Subramanian V, Curcin V, Majeed A, Saxena S: **Impact of early thiopurines on surgery in 2770 children and young people diagnosed with inflammatory bowel disease: a national population-based study**. *Aliment Pharmacol Ther* 2015, **42**:990–999.

45. Coche JC, Colombel JF: **Heterogeneity of inflammatory bowel disease: clinical subgroups of patients**. *Res Clin Forums* 1998, **20**:135–145.

46. Cortot A, Pineton de Chambrun G, Vernier-Massouille G, Vigneron B, Gower-Rousseau C: **Inflammatory bowel disease: Genetic or environmental diseases?. [French] Maladies inflammatoires chroniques de l“intestin : maladies genetiques ou de l”environnement ?** *Gastroenterol Clin Biol* 2009, **33**:681–691.

47. Cosnes J: **Exogenous factors in inflammatory bowel disease**. *Res Clin Forums* 2002, **24**:15–26.

48. Cosnes J: **Tobacco and IBD: relevance in the understanding of disease mechanisms and clinical practice**. *Best Pract Res Clin Gastroenterol* 2004, **18**:481–496.

49. Cosnes J, Beaugerie L, Carbonnel F, Gendre J-P: **Smoking cessation and the course of Crohn's disease: an intervention study**. *Gastroenterology* 2001, **120**:1093–1099.

50. Cosnes J, Carbonnel F, Beaugerie L, Blain A, Reijasse D, Gendre JP: **Effects of appendicectomy on the course of ulcerative colitis**. *Gut* 2002, **51**:803–807.

51. Cosnes J, Carbonnel F, Beaugerie L, Le Quintrec Y, Gendre JP: **Effects of cigarette smoking on the long-term course of Crohn's disease**. *Gastroenterology* 1996, **110**:424–431.

52. Cosnes J, Seksik P: **Environmental factors in Crohn's disease. [French, English]**

**Facteurs environnementaux dans la maladie de Crohn**. *Acta Endoscopica* 2006, **36**:679–688.

53. Cosnes J, Seksik P, Nion-Larmurier I, Beaugerie L, Gendre JP: **Prior appendectomy and the phenotype and course of Crohn's disease**. *World J Gastroenterol* 2006, **12**:1235–1242.

54. Cottone M, Rosselli M, Guinta M, Camma C: **Tobacco and inflammatory bowel disease: epidemiology and influence on IBD evolution**. *Res Clin Forums* 1998, **20**:15–24.

55. Coward S, Heitman SJ, Clement F, Negron M, Panaccione R, Ghosh S, Barkema HW, Seow C, Leung YP, Kaplan GG: **Funding a smoking cessation program for Crohn's disease: an economic evaluation**. *Am J Gastroenterol* 2015, **110**:368–377.

56. Curdia Goncalves T, Dias De Castro F, Machado JF, Moreira MJ, Rosa B, Cotter J: **Impact of the age of diagnosis on the natural history of ulcerative colitis**. *Rev Esp Enferm Dig* 2015, **107**:614–621.

57. Da Silva BC, Lyra AC, Mendes CMC, Ribeiro CPO, Lisboa SRO, De Souza MTL, Portela RC, Santana GO: **The demographic and clinical characteristics of ulcerative colitis in a northeast Brazilian population**. *Biomed Res Int* 2015, **2015**:359130.

58. Danese S, Sans M, Fiocchi C: **Inflammatory bowel disease: the role of environmental factors**. *Autoimmun Rev* 2004, **3**:394–400.

59. Dassopoulos T, Frangakis C, Cruz-Correa M, Talor MV, Burek LC, Datta L, Nouvet F, Bayless TM, Brant SR: **Antibodies to Saccharomyces cerevisiae in Crohnʼs disease**. *Inflamm Bowel Dis* 2007, **13**:143–151.

60. Dassopoulos T, Nguyen GC, Talor MV, Datta LW, Isaacs KL, Lewis JD, Gold MS, Valentine JF, Smoot DT, Harris ML, Oliva-Hemker M, Bayless TM, Consortium NIG, Burek CL, Brant SR: **NOD2 mutations and anti-Saccharomyces cerevisiae antibodies are risk factors for Crohn's disease in African Americans**. *Am J Gastroenterol* 2010, **105**:378–386.

61. De Greef E, Hofman I, Smets F, Van Biervliet S, Scaillon M, Hauser B, Paquot I, Alliet P, Arts W, Dewit O, Peeters H, Baert F, D'Haens G, Rahier JF, Etienne I, Bauraind O, Van Gossum A, Vermeire S, Fontaine F, Muls V, Louis E, Van De Mierop F, Coche JC, Mahachie JJM, Veereman G: **Profile of Belgian pediatric Crohn's disease patients: Presentation and diagnostic features**. *J Crohns Colitis* 2011, **5 (1)**:S155–S156.

62. De Saussure P, Clerson P, Prost PL, Truong Tan N, Bouhnik Y, Gil R: **Appendectomy, smoking habits and the risk of developing ulcerative colitis: A case control study in private practice setting**. *Gastroenterol Clin Biol* 2007, **31**:493–497.

63. Desmond AN, Shanahan F: **Managing chronic disease in Ireland: Hospital admission rates and clinical outcomes in a large ulcerative colitis population**. *Ir J Med Sci* 2012, **181**:65–71.

64. Detry R, Fiasse R: **Role of surgery in the management of Crohn's disease. [French, English] Place de la chirurgie dans le traitement de la maladie de Crohn**. *Acta Endoscopica* 2003, **33**:241–248.

65. Dhar A: **Environmental factors associated with Crohn's disease in India-there's more to it than meets the eye**. *Indian J Gastroenterol* 2011, **30**:255–256.

66. Dias De Castro F, Boal Carvalho P, Monteiro S, Rosa B, Firmino-Machado J, Moreira MJ, Cotter J: **Lewis Score--prognostic value in patients with isolated small bowel Crohn's disease**. *J Crohns Colitis* 2015, **9**:1146–1151.

67. Dobrowolska-Zachwieja A, Kaczmarek M, Hoppe-Golebiewska J, Kalak R, Slomski R, Linke K: **Does smoking influence on the course of Crohn's disease in patients with the presence of gene NOD2/CARD15 mutation?. [Polish] Czy palenie papierosow ma wplyw na przebieg choroby Lesniowskiego-Crohna u chorych z obecnym wariantem mutacji genu NOD2/CARD15?** *Pol Merkur Lekarski* 2005, **18**:560–565.

68. Domoslawski P, Forkasiewicz Z, Firkowska M: **Inflammatory bowel diseases. [Polish] Choroby zapalne jelit**. *Wiad Lek* 1997, **50**:226–229.

69. Elloumi H, Abdelaziz AB, Arfaoui D, Lassoued Y, Letaief R, Jmaa A, Ghannem H, Ajmi S: **Predictive factors of surgery in severe attacks of ulcerative colitis. [French] Facteurs predictifs de recours a la chirurgie dans la rectocolite ulcero hemorragique en poussee grave**. *Tunis Med* 2007, **85**:417–420.

70. Elloumi H, Abdelaziz AB, Derbel F, Jmaa A, Lassoued Y, Arfaoui D, Ali AB, Letaief R, Hamida RBH, Ghannem H, Ajmi S: **Predictive factors of glucocorticosteroid treatment failure in severe acute idiopathic colitis**. *Acta Gastroenterol Belg* 2005, **68**:226–229.

71. Etienney I, Soule JC: **Is a treatment for prevention of relapses always effective in Crohn“s disease? [French] Un traitement d”entretien est-Il toujours utile dans la maladie de crohn?** *Medecine et Hygiene* 2000, **58**:1732–1737.

72. Ferguson LR, Huebner C, Petermann I, Gearry RB, Barclay ML, Demmers P, McCulloch A, Han DH: **Single nucleotide polymorphism in the tumor necrosis factor-alpha gene affects inflammatory bowel diseases risk**. *World J Gastroenterol* 2008, **14**:4652–4661.

73. Fernandez-Blanco Hernaiz JI, Monturiol Jalon JM: **When is much too soon and much too late for surgery in Crohn's disease? [Spanish] ?Cuando es demasiado pronto y cuando demasiado tarde para la cirugia en la enfermedad de Crohn?** *Rev Esp Enferm Dig* 2008, **100**:35–44.

74. Fiocchi C: **Inflammatory bowel disease. Current concepts of pathogenesis and implications for therapy**. *Minerva Gastroenterol Dietol* 2002, **48**:215–226.

75. Fischer S, Lakatos PL, Hungarian IBD Study Group, Lakatos L, Kovacs A, Molnar T, Altorjay I, Papp M, Szilvasi A, Tulassay Z, Osztovits J, Papp J, Demeter P, Schwab R, Tordai A, Andrikovics H: **ATP-binding cassette transporter ABCG2 (BCRP) and ABCB1 (MDR1) variants are not associated with disease susceptibility, disease phenotype response to medical therapy or need for surgery in Hungarian patients with inflammatory bowel diseases**. *Scand J Gastroenterol* 2007, **42**:726–733.

76. Florin THJ, Pandeya N, Radford-Smith GL: **Epidemiology of appendicectomy in primary sclerosing cholangitis and ulcerative colitis: its influence on the clinical behaviour of these diseases**. *Gut* 2004, **53**:973–979.

77. Forcione DG, Rosen MJ, Kisiel JB, Sands BE: **Anti-Saccharomyces cerevisiae antibody (ASCA) positivity is associated with increased risk for early surgery in Crohn's disease**. *Gut* 2004, **53**:1117–1122.

78. Fraga XF, Vergara M, Medina C, Casellas F, Bermejo B, Malagelada JR: **Effects of smoking on the presentation and clinical course of inflammatory bowel disease**. *Eur J Gastroenterol Hepatol* 1997, **9**:683–687.

79. Frisch M: **Inverse association between appendicectomy and ulcerative colitis**. *BMJ* 2006, **332**:561–562.

80. Frolkis AD, Dieleman LA, Barkema HW, Panaccione R, Ghosh S, Fedorak RN, Madsen K, Kaplan GG, Alberta IBD Consortium: **Environment and the inflammatory bowel diseases**. *Can J Gastroenterol* 2013, **27**:e18–e24.

81. Fu YT, Hong T, Round A, Bressler B: **Impact of medical therapy on patients with Crohn's disease requiring surgical resection**. *World J Gastroenterol* 2014, **20**:11808–11814.

82. Gaburri PD, Chebli JM, de Castro LE, Ferreira JO, Lopes MH, Ribeiro AM, Alves RA, Froede EC, de Oliveira KS, Gaburri AK, Gaburri D, Meirelles GS, de Souza AF: **Epidemiologia, aspectos clinicos e evolutivos da doenca de Crohn: um estudo de 60 casos [Epidemiology, clinical features and clinical course of Crohn's disease: a study of 60 cases]**. *Arq Gastroenterol* 1998, **35**:240–246.

83. Gapasin J, Van Langenberg DR, Holtmann G, Hetzel DJ, Andrews JM: **Potentially avoidable surgery in inflammatory bowel disease: What proportion of patients come to resection without optimal preoperative therapy? A guidelines-based audit**. *Intern Med J* 2012, **42**:e84–e88.

84. Garcia Rodriguez LA, Gonzalez-Perez A, Johansson S, Wallander MA: **Risk factors for inflammatory bowel disease in the general population**. *Aliment Pharmacol Ther* 2005, **22**:309–315.

85. García Paredes J, Mendoza JL: **Epidemiologia descriptiva de la enfermedad inflamatoria intestinal en Espana [Descriptive epidemiology of inflammatory bowel disease in Spain]**. *An Med Interna* 2003, **20**:1–2.

86. Garrido Serrano A, Martinez MJ, Ortega JA, Lobato A, Rodriguez MJ, Guerrero FJ: **Estudio epidemiologico de la enfermedad inflamatoria intestinal en la zona norte de Huelva [Epidemiology of chronic inflammatory bowel disease in the Northern area of Huelva]**. *Rev Esp Enferm Dig* 2004, **96**:687–694.

87. Gathungu G, Kim MO, Ferguson JP, Sharma Y, Zhang W, Ng SM, Bonkowski E, Ning K, Simms LA, Croft AR, Stempak JM, Walker N, Huang N, Xiao Y, Silverberg MS, Trapnell B, Cho JH, Radford-Smith GL, Denson LA: **Granulocyte-macrophage colony-stimulating factor autoantibodies: a marker of aggressive Crohn's disease**. *Inflamm Bowel Dis* 2013, **19**:1671–1680.

88. Goischke HK: **Tabakkonsum und morbus Crohn: anlass zur therapeutischen herausforderung oder berechtigter nihilismus? [Tobacco consumption and Crohn's disease - reason for a selective treatment or legitimate nihilism?]**. *Med Welt* 2003, **54**:185–189.

89. Goldberg PA, Wright JP, Gerber M, Claassen R: **Incidence of surgical resection for Crohn's disease**. *Dis Colon Rectum* 1993, **36**:736–739.

90. Golovics PA, Lakatos L, Nagy A, Pandur T, Szita I, Balogh M, Molnar C, Komaromi E, Lovasz BD, Mandel M, Veres G, Kiss LS, Vegh Z, Lakatos PL: **Is early limited surgery associated with a more benign disease course in Crohn's disease?** *World J Gastroenterol* 2013, **19**:7701–7710.

91. Guo AY, Stevens BW, Wilson RG, Russell CN, Cohen MA, Sturgeon HC, Thornton A, Giallourakis C, Khalili H, Nguyen DD, Sauk J, Yajnik V, Xavier RJ, Ananthakrishnan AN: **Early life environment and natural history of inflammatory bowel diseases**. *BMC Gastroenterol* 2014, **14**:216.

92. Gustavsson A, Magnuson A, Blomberg B, Andersson M, Halfvarson J, Tysk C: **Smoking is a risk factor for recurrence of intestinal stricture after endoscopic dilation in Crohn's disease**. *Aliment Pharmacol Ther* 2013, **37**:430–437.

93. Halfvarson J, Jess T, Magnuson A, Montgomery SM, Orholm M, Tysk C, Binder V, Jarnerot G: **Environmental factors in inflammatory bowel disease: A co-twin control study of a Swedish-Danish twin population**. *Inflamm Bowel Dis* 2006, **12**:925–933.

94. Hanauer SB: **Nicotine for colitis - the smoke has not yet cleared**. *N Engl J Med* 1994, **330**:856–857.

95. Hanauer SB: **Disease modification in inflammatory bowel disease**. *Clin Gastroenterol Hepatol* 2012, **10**:954–955.

96. Hanauer SB, Present DH: **Avoiding common errors in maintenance therapy for ulcerative colitis**. *Pract Gastroenterol* 1998, **22**:49–58.

97. Hancock L, Beckly J, Geremia A, Cooney R, Cummings F, Pathan S, Guo C, Warren BF, Mortensen N, Ahmad T, Jewell D: **Clinical and molecular characteristics of isolated colonic Crohn's disease**. *Inflamm Bowel Dis* 2008, **14**:1667–1677.

98. Hansen TS, Jess T, Vind I, Elkjaer M, Nielsen MF, Gamborg M, Munkholm P: **Environmental factors in inflammatory bowel disease: a case-control study based on a Danish inception cohort**. *J Crohns Colitis* 2011, **5**:577–584.

99. Harper JW, Welch MP, Sinanan MN, Wahbeh GT, Lee SD: **Co-morbid diabetes in patients with Crohn's disease predicts a greater need for surgical intervention**. *Aliment Pharmacol Ther* 2012, **35**:126–132.

100. Hatch Q, Champagne BJ, Maykel JA, Davis BR, Johnson EK, Bleier JI, Francone TD, Steele SR: **The impact of pregnancy on surgical Crohn disease: An analysis of the Nationwide Inpatient Sample**. *J Surg Res* 2014, **190**:41–46.

101. Henriksen M, Jahnsen J, Lygren I, Vatn MH, Moum B, Ibsen Study Group: **Are there any differences in phenotype or disease course between familial and sporadic cases of inflammatory bowel disease? Results of a population-based follow-up study**. *Am J Gastroenterol* 2007, **102**:1955–1963.

102. Hoie O, Aamodt G, Vermeire S, Bernklev T, Odes S, Wolters FL, Riis L, Politi P, Tsianos EV, Butron M, Stockbrugger RW, Munkholm P, Vatn M, Moum B: **Serological markers are associated with disease course in ulcerative colitis. A study in an unselected population-based cohort followed for 10 years**. *J Crohns Colitis* 2008, **2**:114–122.

103. Hoie O, Wolters F, Riis L, Aamodt G, Solberg C, Bernklev T, Odes S, Mouzas IA, Beltrami M, Langholz E, Stockbrugger R, Vatn M, Moum B: **Ulcerative colitis: patient characteristics may predict 10-yr disease recurrence in a European-wide population-based cohort**. *Am J Gastroenterol* 2007, **102**:1692–1701.

104. Hovde O, Moum BA: **Epidemiology and clinical course of Crohn's disease: results from observational studies**. *World J Gastroenterol* 2012, **18**:1723–1731.

105. Jiang XL, Cui HF: **An analysis of 10218 ulcerative colitis cases in China**. *World J Gastroenterol* 2002, **8**:158–161.

106. Jiang XL, Gui HF: **Features of ulcerative colitis in china: An analysis 10 218 cases. [Chinese]**. *World Chinese Journal of Digestology* 2001, **9**:869–873.

107. Kalkan IH, Dagli U, Oztas E, Tunc B, Ulker A: **Comparison of demographic and clinical characteristics of patients with early vs. adult vs. late onset ulcerative colitis**. *Eur J Intern Med* 2013, **24**:273–277.

108. Kanneganti M, Dehghani B, Steinberg J, Cerda S, Rybin D, Weinberg J, Farraye FA: **Significance of granulomatous inflammation found on endoscopic biopsies or surgical resections on the severity of Crohn's disease**. *J Clin Gastroenterol* 2013, **47**:894.

109. Kaplan GG, Hubbard J, Korzenik J, Sands BE, Panaccione R, Ghosh S, Wheeler AJ, Villeneuve PJ: **The inflammatory bowel diseases and ambient air pollution: a novel association**. *Am J Gastroenterol* 2010, **105**:2412–2419.

110. Kaplan GG, Seow CH, Ghosh S, Molodecky N, Rezaie A, Moran GW, Proulx MC, Hubbard J, MacLean A, Buie D, Panaccione R: **Decreasing colectomy rates for ulcerative colitis: a population-based time trend study**. *Am J Gastroenterol* 2012, **107**:1879–1887.

111. Karczewski J, Poniedzialek B, Piotr R, Malgorzata M: **Different effect of smoking on genders in Crohn's disease**. *Turk J Gastroenterol* 2015, **26**:133–139.

112. Karczewski J, Poniedzialek B, Rzymski P, Rychlewska-Hanczewska A, Adamski Z, Wiktorowicz K: **The effect of cigarette smoking on the clinical course of inflammatory bowel disease**. *Prz Gastroenterol* 2014, **9**:153–159.

113. Kennelly RP, Subramaniam T, Egan LJ, Joyce M: **Smoking and Crohn's disease: active modification of an independent risk factor (education alone is not enough)**. *J Crohns Colitis* 2013, **7**:631–635.

114. Kim YJ: **Risk factors for repeat abdominal surgery in patients with Crohn's disease**. *J Korean Soc Coloproctol* 2012, **28**:175.

115. Kiss LS, Papp M, Lovasz BD, Vegh Z, Golovics PA, Janka E, Varga E, Szathmari M, Lakatos PL: **High-sensitivity C-reactive protein for identification of disease phenotype, active disease, and clinical relapses in Crohn's disease: a marker for patient classification?** *Inflamm Bowel Dis* 2012, **18**:1647–1654.

116. Koutroubakis I, Manousos ON, Meuwissen SGM, Peña AS: **Environmental risk factors in inflammatory bowel disease**. *Hepatogastroenterology* 1996, **43**:381–393.

117. Krishnan A, Korzenik JR: **Inflammatory bowel disease and environmental influences**. *Gastroenterol Clin North Am* 2002, **31**:21–39.

118. Laghi L, Costa S, Saibeni S, Bianchi P, Omodei P, Carrara A, Spina L, Contessini Avesani E, Vecchi M, De Franchis R, Malesci A: **Carriage of CARD15 variants and smoking as risk factors for resective surgery in patients with Crohn's ileal disease**. *Aliment Pharmacol Ther* 2005, **22**:557–564.

119. Lakatos PL, Lakatos L, Szalay F, Willheim-Polli C, Osterreicher C, Tulassay Z, Molnar T, Reinisch W, Papp J, Mozsik G, Ferenci P, Fuszek P, Vargha P, Pronai L, Nemeth A, Miheller P, Kovacs A, Bene L, Szekely G, Gasztonyi B, Nagy F, Lonovics J, Ujszaszy L, Altorjai I, Palatka K, Kiss GG, Tarnok F, Dobronte Z, Erdelyi Z, Pandur T, et al.: **Toll-like receptor 4 and NOD2/CARD15 mutations in Hungarian patients with Crohn's disease: phenotype-genotype correlations**. *World J Gastroenterol* 2005, **11**:1489–1495.

120. Lakatos PL, Szalay F, Tulassay Z, Molnar T, Kovacs A, Gasztonyi B, Papp J, Lakatos L: **Clinical presentation of Crohn's disease. Association between familial disease, smoking, disease phenotype, extraintestinal manifestations and need for surgery**. *Hepatogastroenterology* 2005, **52**:817–822.

121. Lakatos PL: **Environmental factors affecting inflammatory bowel disease: Have we made progress?** *Dig Dis* 2009, **27**:215–225.

122. Lakatos PL, Szamosi T, Lakatos L: **Smoking in inflammatory bowel diseases: good, bad or ugly?** *World J Gastroenterol* 2007, **13**:6134–6139.

123. Lakatos PL, Vegh Z, Lovasz BD, David G, Pandur T, Erdelyi Z, Szita I, Mester G, Balogh M, Szipocs I, Molnar C, Komaromi E, Golovics PA, Mandel M, Horvath A, Szathmari M, Kiss LS, Lakatos L: **Is current smoking still an important environmental factor in inflammatory bowel diseases? Results from a population-based incident cohort**. *Inflamm Bowel Dis* 2013, **19**:1010–1017.

124. Law ST, Li KK: **Age-related differences in the clinical course of Crohn's disease in an Asian population: a retrospective cohort review**. *Indian Pediatr* 2013, **50**:1148–1152.

125. Lazarev M, Huang C, Bitton A, Cho JH, Duerr RH, McGovern DP, Proctor DD, Regueiro M, Rioux JD, Schumm PP, Taylor KD, Silverberg MS, Hillary Steinhart A, Hutfless S, Brant SR: **Relationship between proximal Crohn's disease location and disease behavior and surgery: a cross-sectional study of the IBD genetics consortium**. *Am J Gastroenterol* 2013, **108**:106–112.

126. Leiss O, Borner N, Clement T, Godderz W, Schreiber HJ, Lutz-Vorderbrugge A: **[Ernahrungsfaktoren in genese und therapie chronisch-entzundlicher darmerkrankungen (Morbus Crohn und colitis ulcerosa) [Nutrition in pathogenesis and management of inflammatory bowel disease (Crohn's disease and ulcerative colitis)]**. *Verdauungskrankheiten* 2004, **22**:319–337.

127. Leong RW, Lawrance IC, Chow DK, To KF, Lau JY, Wu J, Leung WK, Chan FK, Sung JJ: **Association of intestinal granulomas with smoking, phenotype, and serology in Chinese patients with Crohn's disease**. *Am J Gastroenterol* 2006, **101**:1024–1029.

128. Leong RWL: **Environmental risk factors in inflammatory bowel disease**. *J Gastroenterol Hepatol* 2010, **25**:227–228.

129. Lunney PC, Leong RWL: **Review article: ulcerative colitis, smoking and nicotine therapy**. *Aliment Pharmacol Ther* 2012, **36**:997–1008.

130. Lunney PC, Kariyawasam VC, Wang RR, Middleton KL, Huang T, Selinger CP, Andrews JM, Katelaris PH, Leong RW: **Smoking prevalence and its influence on disease course and surgery in Crohn's disease and ulcerative colitis**. *Aliment Pharmacol Ther* 2015, **42**:61–70.

131. Manser CN, Mecklenburg I, Knellwolf C, Meyenberger C, Seibold F, Muenger-Beyeler C, Biedermann L, Vavricka S, Frei P, Rogler G: **Risk factors for complications in ulcerative colitis patients**. *J Crohns Colitis* 2013, **7**:S70–S71.

132. Manser CN, Borovicka J, Seibold F, Vavricka SR, Lakatos PL, Fried M, Rogler G, investigators of the Swiss Inflammatory Bowel Disease Cohort S: **Risk factors for complications in patients with ulcerative colitis**. *United European Gastroenterol* 2016, **4**:281–287.

133. Mao R, Chen BL, He Y, Cui Y, Zeng ZR, Chen MH: **Factors associated with progression to surgery in Crohn's disease patients with endoscopic stricture**. *Endoscopy* 2014, **46**:956–962.

134. Margagnoni G, Fasci Spurio F, Feigusch L, Koch M, Papi C, Aratari A: **Long term course of ulcerative colitis in the prebiologic era. A retrospective study in a tertiary referral center**. *Minerva Gastroenterol Dietol* 2014, **60**:275–283.

135. Mazor Y, Maza I, Kaufman E, Ben-Horin S, Karban A, Chowers Y, Eliakim R: **Prediction of disease complication occurrence in Crohn's disease using phenotype and genotype parameters at diagnosis**. *J Crohns Colitis* 2011, **5**:592–597.

136. McDermott E, Murphy S, Keegan D, O'Donoghue D, Mulcahy H, Doherty G: **Efficacy of adalimumab as a long term maintenance therapy in ulcerative colitis**. *J Crohns Colitis* 2013, **7**:150–153.

137. Medina C, Vergara M, Casellas F, Lara F, Naval J, Malagelada JR: **Influence of the smoking habit in the surgery of inflammatory bowel disease**. *Rev Esp Enferm Dig* 1998, **90**:771–778.

138. Mokbel M, Carbonnel F, Beaugerie L, Gendre JP, Cosnes J: **Effet du tabac sur l'evolution long terme de la rectocolite hemorragique [Effect of smoking on the long-term course of ulcerative colitis]**. *Gastroenterol Clin Biol* 1998, **22**:858–862.

139. Monterubbianesi R, Aratari A, Armuzzi A, Daperno M, Biancone L, Cappello M, Annese V, Riegler G, Orlando A, Viscido A, Meucci G, Gasbarrini A, Guidi L, Lavagna A, Sostegni R, Onali S, Papi C, Kohn A: **Infliximab three-dose induction regimen in severe corticosteroid-refractory ulcerative colitis: early and late outcome and predictors of colectomy**. *J Crohns Colitis* 2014, **8**:852–858.

140. Morgan AR, Han DY, Huebner C, Lam WJ, Fraser AG, Ferguson LR: **PTPN2 but not PTPN22 is associated with Crohn's disease in a New Zealand population**. *Tissue Antigens* 2010, **76**:119–125.

141. Moum B, Ekbom A, Vatn MH, Aadland E, Sauar J, Lygren I, Schulz T, Stray N, Fausa O: **Clinical course during the 1st year after diagnosis in ulcerative colitis and Crohn's disease: results of a large, prospective population- based study in southeastern Norway, 1990-93**. *Scand J Gastroenterol* 1997, **32**:1005–1012.

142. Mozdiak E, O'Malley J, Arasaradnam R: **Inflammatory bowel disease**. *BMJ* 2015, **351**:h4416.

143. Nasir BF, Griffiths L, Nasir A, Roberts R, Barclay M, Gearry R, Lea RA: **Perianal disease combined with NOD2 genotype predicts need for IBD-related surgery in Crohn's disease patients from a population-based cohort**. *J Clin Gastroenterol* 2013, **47**:242–245.

144. Nasir BF, Griffiths LR, Nasir A, Roberts R, Barclay M, Gearry RB, Lea RA: **An envirogenomic signature is associated with risk of IBD-related surgery in a population-based Crohn's disease cohort**. *J Gastrointest Surg* 2013, **17**:1643–1650.

145. Ng SC, Woodrow S, Patel N, Subhani J, Harbord M: **Role of genetic and environmental factors in British twins with inflammatory bowel disease**. *Inflamm Bowel Dis* 2012, **18**:725–736.

146. Nuij V, Fuhler GM, Edel AJ, Ouwendijk RJ, Rijk MC, Beukers R, Quispel R, van Tilburg AJ, Tang TJ, Smalbraak H, Bruin KF, Lindenburg F, Peyrin-Biroulet L, Van Der Woude CJ, Dutch Delta IBDG: **Benefit of earlier anti-TNF treatment on IBD disease complications?** *J Crohns Colitis* 2015, **9**:997–1003.

147. Onal IK: **Effect of steroid sparing agents on the clinical course of ulcerative colitis**. *Eur J Intern Med* 2014, **25**:e19.

148. Onali S, Petruzziello C, Calabrese E, Condino G, Zorzi F, Sica GS, Pallone F, Biancone L: **Frequency, pattern, and risk factors of postoperative recurrence of Crohn's disease after resection different from ileo-colonic**. *J Gastrointest Surg* 2009, **13**:246–252.

149. Parkes GC, Whelan K, Lindsay JO: **Smoking in inflammatory bowel disease: Impact on disease course and insights into the aetiology of its effect**. *J Crohns Colitis* 2014, **8**:717–725.

150. Perri F, Annese V, Napolitano G, Caruso N, Clemente R, Villani MR, Andriulli A: **Subgroups of patients with Crohn's disease have different clinical outcomes**. *Inflamm Bowel Dis* 1996, **2**:1–5.

151. Picco MF, Zubiaurre I, Adluni M, Cangemi JR, Shelton D: **Immunomodulators are associated with a lower risk of first surgery among patients with non-penetrating non-stricturing Crohn's disease**. *Am J Gastroenterol* 2009, **104**:2754–2759.

152. Pittet V, Rogler G, Michetti P, Fournier N, Vader JP, Schoepfer A, Mottet C, Burnand B, Froehlich F: **Penetrating or stricturing diseases are the major determinants of time to first and repeat resection surgery in Crohn's disease for the Swiss inflammatory bowel disease cohort study group**. *Digestion* 2013, **87**:212–221.

153. Prosberg MV, Vester-Andersen MK, Andersson M, Jess T, Andersen JT, Vind I, Bendtsen F: **Long-term compliance with oral 5-aminosalicylic acid therapy and risk of disease recurrence in patients with ulcerative colitis: A population-based cohort study**. *Inflamm Bowel Dis* 2016, **22**:925–932.

154. Puspok A, Dejaco C, Oberhuber G, Waldhor T, Hirschl AM, Vogelsang H, Gasche C: **Influence of Helicobacter pylori infection on the phenotype of Crohn's disease**. *Am J Gastroenterol* 1999, **94**:3239–3244.

155. Reenaers C, Pirard C, Vankemseke C, Latour P, Belaiche J, Louis E: **Long-term evolution and predictive factors of mild inflammatory bowel disease**. *Scand J Gastroenterol* 2016, **51**:712–719.

156. Regueiro M, Kip KE, Baidoo L, Swoger JM, Schraut W: **Reply**. *Clin Gastroenterol Hepatol* 2014, **12**:1764–1765.

157. Rentsch M, Beham A, Schlitt HJ, Jauch KW: **Crohn's disease activity index and Vienna classification - is it worthwhile to calculate before surgery?** *Dig Surg* 2006, **23**:241–249.

158. Ricciardi R: **Does cigarette smoking influence the phenotype of Crohn's disease? Analysis using the Montreal Classification**. *Dis Colon Rectum* 2007, **50**:1288–1289.

159. Riegler G, Caserta L, Esposito I, De Filippo FR, Bossa F, Esposito P, Russo MI, Carratu R: **Worse clinical course of disease in Crohn's patients with previous appendectomy**. *Eur J Gastroenterol Hepatol* 2005, **17**:623–627.

160. Ritzmann P: **Chronisch-entzundliche darmkrankheiten [Chronic inflammatory bowel diseases]**. *Pharma-Kritik* 2003, **25**:65–68.

161. Romberg-Camps MJ, Dagnelie, P. C., Kester AD, Hesselink-van de Kruijs MA, Cilissen M, Engels LG, Van Deursen C, Hameeteman WH, Wolters FL, Russel MG, Stockbrugger RW: **Influence of phenotype at diagnosis and of other potential prognostic factors on the course of inflammatory bowel disease**. *Am J Gastroenterol* 2009, **104**:371–383.

162. Russel MG, Volovics A, Schoon EJ, van Wijlick EH, Logan RF, Shivananda S, Stockbrugger RW: **Inflammatory bowel disease: is there any relation between smoking status and disease presentation? European Collaborative IBD Study Group**. 1998, **4**:182–186.

163. Ryan WR, Ley C, Allan RN, Keighley MR: **Patients with Crohn's disease are unaware of the risks that smoking has on their disease**. *J Gastrointest Surg* 2003, **7**:706–711.

164. Safroneeva E, Vavricka SR, Fournier N, Straumann A, Rogler G, Schoepfer AM: **Prevalence and risk factors for therapy escalation in ulcerative colitis in the Swiss IBD Cohort Study**. *Inflamm Bowel Dis* 2015, **21**:1348–1358.

165. Sands B: **Risk of early surgery for Crohn's disease: implications for early treatment strategies**. *Am J Gastroenterol* 2003, **98**:2712–2718.

166. Sato Y, Matsui T, Yano Y, Tsurumi K, Okado Y, Matsushima Y, Koga A, Takahashi H, Ninomiya K, Ono Y, Takatsu N, Beppu T, Nagahama T, Hisabe T, Takaki Y, Hirai F, Yao K, Higashi D, Futami K, Washio M: **Long-term course of Crohn's disease in Japan: incidence of complications, cumulative rate of initial surgery, and risk factors at diagnosis for initial surgery**. *J Gastroenterol Hepatol* 2015, **30**:1713–1719.

167. Satsangi J, Grootscholten C, Holt H, Jewell DP: **Clinical patterns of familial inflammatory bowel disease**. *Gut* 1996, **38**:738–741.

168. Savoye G, Lerebours E: **Toll-like receptor-4 signaling: A possible candidate pathway to support tobacco smoking effects in ulcerative colitis**. *Am J Gastroenterol* 2008, **103**:2947–2948.

169. Seksik P, Nion-Larmurier I, Sokol H, Beaugerie L, Cosnes J: **Effects of light smoking consumption on the clinical course of Crohn's disease**. *Dis Colon Rectum* 2009, **52**:1678–1679.

170. Seksik P, Nion-Larmurier I, Sokol H, Beaugerie L, Cosnes J: **Effects of light smoking consumption on the clinical course of Crohn's disease**. *Inflamm Bowel Dis* 2009, **15**:734–741.

171. Sercombe J: **Inflammatory bowel disease and smoking**. *Prof Nurse* 2000, **15**:439–442.

172. Sharma A, Deeb AP, Iannuzzi JC, Rickles AS, Monson JR, Fleming FJ: **Tobacco smoking and postoperative outcomes after colorectal surgery**. *Ann Surg* 2013, **258**:296–300.

173. Sicilia B, Vicente R, Arroyo MT, Arribas F, Gomollon F: **Cirugia de una cohorte incidente de pacientes con enfermedad de Crohn en Aragon: Indicaciones, tipo de cirugia y factores de riesgo asociados [Surgery at follow-up in an incidence cohort of patients with Crohn's disease in Aragon (Spain): Etiology, type of surgery and associated epidemiological factors]**. *Gastroenterol Hepatol* 2005, **28**:105–109.

174. Sicilia B, Vicente R, Arroyo MT, Arribas F, Gomollon F: **La afectacion pancolonica inicial en la colitis ulcerosa predice la necesidad de colectomia: Estudio poblacional basado en una cohorte incidente de pacientes en Aragon [Ulcerative pancolitis predicts the need for colectomy: Study of an incident cohort of patients with ulcerative colitis in Aragon (Spain)]**. *Gastroenterol Hepatol* 2005, **28**:55–59.

175. Siddique I, Alazmi W, Al-Ali J, Longenecker JC, Al-Fadli A, Hasan F, Memon A: **Demography and clinical course of ulcerative colitis in Arabs - a study based on the Montreal classification**. *Scand J Gastroenterol* 2014, **49**:1432–1440.

176. Slattery E, Keegan D, Hyland J, O'Donoghue D, Mulcahy HE: **Surgery, Crohn's disease, and the biological era: has there been an impact?** *J Clin Gastroenterol* 2011, **45**:691–693.

177. Smith BRK, Arnott IDR, Drummond HE, Nimmo ER, Satsangi J: **Disease location, anti-Saccharomyces cerevisiae antibody, and NOD2/CARD15 genotype influence the progression of disease behavior in Crohn's disease**. *Inflamm Bowel Dis* 2004, **10**:521–528.

178. Solberg IC, Hoivik ML, Cvancarova M, Moum B, Vatn M, Lygren I, Jahnsen J, Hoie O, Henriksen M, Stray N, Bernklev T, Sauar J: **Risk matrix model for prediction of colectomy in a population-based study of ulcerative colitis patients (the IBSEN study)**. *Scand J Gastroenterol* 2015, **50**:1456–1462.

179. Song XM, Gao X, Li MZ, Chen ZH, Chen SC, Hu PJ, He YL, Zhan WH, Chen MH: **Clinical features and risk factors for primary Surgery in 205 patients with Crohn's disease: analysis of a South China cohort**. *Dis Colon Rectum* 2011, **54**:1147–1154.

180. Storr M, Devlin S, Kaplan GG, Panaccione R, Andrews CN: **Cannabis use provides symptom relief in patients with inflammatory bowel disease but is associated with worse disease prognosis in patients with Crohn's disease**. *Inflamm Bowel Dis* 2014, **20**:472–480.

181. Strong S, Steele SR, Boutrous M, Bordineau L, Chun J, Stewart DB, Vogel J, Rafferty JF: **Clinical practice guideline for the surgical management of Crohn's disease**. *Dis Colon Rectum* 2015, **58**:1021–1036.

182. Szamosi T, Banai J, Lakatos L, Czegledi Z, David G, Zsigmond F, Pandur T, Erdelyi Z, Gemela O, Papp M, Papp J, Lakatos PL: **Early azathioprine/biological therapy is associated with decreased risk for first surgery and delays time to surgery but not reoperation in both smokers and nonsmokers with Crohn's disease, while smoking decreases the risk of colectomy in ulcerative colitis**. *Eur J Gastroenterol Hepatol* 2010, **22**:872–879.

183. Szymanska S, Lördal M, Rathnayake N, Gustafsson A, Johannsen A: **Dental caries, prevalence and risk factors in patients with Crohn's disease**. *PLoS One* 2014, **9**:e91059.

184. Taxonera C, Estelles J, Fernandez-Blanco I, Merino O, Marin-Jimenez I, Barreiro-De Acosta M, Saro C, Garcia-Sanchez V, Gento E, Bastida G, Gisbert JP, Vera I, Martinez-Montiel P, Garcia-Moran S, Sanchez MC, Mendoza JL: **Adalimumab induction and maintenance therapy for patients with ulcerative colitis previously treated with infliximab**. *Aliment Pharmacol Ther* 2011, **33**:340–348.

185. Thapa SD, Hadid H, Usman M, Imam W, Hassan A, Schairer J, Jafri SM, Kaur N: **Predictors of thiopurine treatment failure in biologic-naive ulcerative colitis patients**. *Dig Dis Sci* 2016, **61**:230–237.

186. Torres EA, Cruz A, Monagas M, Bernal M, Correa Y, Cordero R, Carlo VL: **Inflammatory bowel disease in Hispanics: the University of Puerto Rico IBD Registry**. *Int J Inflamm* 2012, **2012**:574079–5.

187. Tremaine WJ, Timmons LJ, Loftus EV Jr, Pardi DS, Sandborn WJ, Harmsen WS, Thapa P, Zinsmeister AR: **Age at onset of inflammatory bowel disease and the risk of surgery for non-neoplastic bowel disease**. *Aliment Pharmacol Ther* 2007, **25**:1435–1441.

188. Ungar B, Kopylov U, Goitein D, Lahat A, Bardan E, Avidan B, Lang A, Maor Y, Eliakim R, Ben-Horin S: **Severe and morbid obesity in Crohn's disease patients: prevalence and disease associations**. *Digestion* 2013, **88**:26–32.

189. Van Assche G, D'Haens G, Noman M, Vermeire S, Hiele M, Asnong K, Arts J, D'Hoore A, Penninckx F, Rutgeerts P: **Randomized, double-blind comparison of 4 mg/kg versus 2 mg/kg intravenous cyclosporine in severe ulcerative colitis**. *Gastroenterology* 2003, **125**:1025–1031.

190. Van Der Heide F, Dijkstra A, Weersma RK, Albersnagel FA, van der Logt EMJ, Faber KN, Sluiter WJ, Kleibeuker JH, Dijkstra G: **Effects of active and passive smoking on disease course of Crohn's disease and ulcerative colitis**. *Inflamm Bowel Dis* 2009, **15**:1199–1207.

191. Vander Cruyssen B, Peeters H, Hoffman IEA, Laukens D, Coucke P, Marichal D, Cuvelier C, Remaut E, Veys EM, Mielants H, De Vos M, De Keyser F: **CARD15 polymorphisms are associated with anti-Saccharomyces cerevisiae antibodies in caucasian Crohn's disease patients**. *Clin Exp Immunol* 2005, **140**:354–359.

192. Vecchi M, Saibeni S, De Franchis R: **Risk factors and subclinical markers of inflammatory bowel diseases**. *EOS* 2003, **23**:30–34.

193. Vegh Z, Burisch J, Pedersen N, Kaimakliotis I, Duricova D, Bortlik M, Avnstrom S, Vinding KK, Olsen J, Nielsen KR, Katsanos KH, Tsianos EV, Lakatos L, Schwartz D, Odes S, Lupinacci G, De Padova A, Jonaitis L, Kupcinskas L, Turcan S, Tighineanu O, Mihu I, Barros LF, Magro F, Lazar D, Goldis A, Fernandez A, Hernandez V, Niewiadomski O, Bell S, et al.: **Incidence and initial disease course of inflammatory bowel diseases in 2011 in Europe and Australia: results of the 2011 ECCO-EpiCom inception cohort**. *J Crohns Colitis* 2014, **8**:1506–1515.

194. Vester-Andersen MK, Vind I, Prosberg MV, Bengtsson BG, Blixt T, Munkholm P, Andersson M, Jess T, Bendtsen F: **Hospitalisation, surgical and medical recurrence rates in inflammatory bowel disease 2003-2011-a Danish population-based cohort study**. *J Crohns Colitis* 2014, **8**:1675–1683.

195. Vind I, Riis L, Jess T, Knudsen E, Pedersen N, Elkjaer M, Andersen IB, Wewer V, Norregaard P, Moesgaard F, Bendtsen F, Munkholm P: **Increasing incidences of inflammatory bowel disease and decreasing surgery rates in Copenhagen City and County, 2003-2005: a population-based study from the Danish Crohn colitis database**. *Am J Gastroenterol* 2006, **101**:1274–1282.

196. Vohra P: **Inflammatory bowel disease**. *Indian J Pediatr* 2000, **67**:747–756.

197. Wang MH, Fiocchi C, Zhu X, Ripke S, Kamboh MI, Rebert N, Duerr RH, Achkar JP: **Gene-gene and gene-environment interactions in ulcerative colitis**. *Hum Genet* 2014, **133**:547–558.

198. Waterman M, Knight J, Dinani A, Xu W, Stempak JM, Croitoru K, Nguyen GC, Cohen Z, McLeod RS, Greenberg GR, Steinhart AH, Silverberg MS: **Predictors of outcome in ulcerative colitis**. 2015, **21**:2097–2105.

199. Yamamoto T, Allan RN, Keighley MRB: **Smoking is a predictive factor for outcome after colectomy and ileorectal anastomosis in patients with Crohn's colitis**. *Br J Surg* 1999, **86**:1069–1070.

200. Yamamoto T, Keighley MR: **The association of cigarette smoking with a high risk of recurrence after ileocolonic resection for ileocecal Crohn's disease**. *Surg Today* 1999, **29**:579–580.

201. Yang P, Tremaine WJ, Meyer RL, Prakash UB: **Alpha1-antitrypsin deficiency and inflammatory bowel diseases**. *Mayo Clin Proc* 2000, **75**:450–455.

202. Yarur AJ, Strobel SG, Deshpande AR, Abreu MT: **Predictors of aggressive inflammatory bowel disease**. *Gastroenterol Hepatol (N Y)* 2011, **7**:652–659.

203. Zabana Y, Garcia-Planella E, Van Domselaar M, Manosa M, Gordillo J, Lopez San Roman A, Cabre E, Domenech E: **Does active smoking really influence the course of Crohn's disease? A retrospective observational study**. *J Crohns Colitis* 2013, **7**:280–285.

204. Zabana Y, Garcia-Planella E, van Domselaar M, Manosa M, Gordillo J, Lopez-Sanroman A, Cabre E, Domenech E: **Predictors of favourable outcome in inflammatory Crohn's disease. A retrospective observational study**. *Gastroenterol Hepatol* 2013, **36**:616–623.

205. Zaharie R, Zaharie F, Mocan L, Andreica V, Tantau M, Zdrehus C, Iancu C, Tomus C: **Surgical outcome of inflammatory bowel disease - experience of a tertiary center**. *Chirurgia (Bucur)* 2013, **108**:812–815.

206. Zaharie R, Tantau A, Zaharie F, Tantau M, Gheorghe L, Gheorghe C, Gologan S, Cijevschi C, Trifan A, Dobru D, Goldis A, Constantinescu G, Iacob R, Diculescu M, Group IS: **Diagnostic delay in Romanian patients with inflammatory bowel disease: risk factors and impact on the disease course and need for surgery**. *J Crohns Colitis* 2016, **10**:306–314.

207. Zallot C, Peyrin-Biroulet L: **Clinical risk factors for complicated disease: how reliable are they?** *Dig Dis* 2012, **30**:67–72.

208. Zvidi I, Fraser GM, Niv Y, Birkenfeld S: **The prevalence of inflammatory bowel disease in an Israeli Arab population**. *J Crohns Colitis* 2013, **7**:e159–e163.
